# New insights from the application of ZooMS to Late Pleistocene fauna from Grotta di Castelcivita, southern Italy

**DOI:** 10.1038/s41598-025-11355-6

**Published:** 2025-07-17

**Authors:** Annette Oertle, Jacopo Crezzini, Adriana Moroni, Annamaria Ronchitelli, Stefano Benazzi, Armando Falcucci, Giulia Marciani, Matteo Rossini, Ivan Martini, Simona Arrighi, Tom Higham, Francesco Boschin, Katerina Douka

**Affiliations:** 1 Department of Evolutionary Anthropology, Faculty of Life Sciences, University of Vienna, Vienna, Austria; 2 Human Evolution and Archaeological Sciences (HEAS), University of Vienna, Vienna, Austria; 3 Department of Physical Sciences, Earth and Environment, University of Siena, Siena, Italy; 4 Department of Cultural Heritage, University of Bologna, Bologna, Italy; 5 Department of Geosciences, Prehistory and Archaeological Sciences Research Unit, Eberhard Karls University of Tübingen, Tübingen, Germany

**Keywords:** Zooarchaeology by mass spectrometry (ZooMS), Zooarchaeology, Paleolithic assemblage, Climate-change adaptation, Archaeology

## Abstract

The Middle to Upper Paleolithic cave site of Grotta di Castelcivita (Campania, Southern Italy) contains a key archaeological sequence exhibiting Late Mousterian occupation followed by the Uluzzian techno-complex and an Aurignacian sequence (Protoaurignacian and Early Aurignacian). Abundant faunal remains are found throughout the sequence with variations in taxa present in each period. Previous studies of the morphologically identifiable faunal remains have provided valuable information on species abundance and diversity to reconstruct subsistence behaviour. However, like in many Pleistocene sequences, much of the faunal assemblage is fragmented and unidentifiable. Here we focus on these unidentified fragmentary bones to add greater dimension to the observed patterns. The application of collagen peptide mass fingerprinting (or Zooarchaeology by Mass Spectrometry; ZooMS) on 1263 unidentified bones revealed distinct changes in ZooMS NISP values in the Uluzzian and Protoaurignacian periods where equids and bovids nearly doubled in quantity compared to the original morphologically identified macrofaunal assemblage. New ZooMS identifications of rhinoceros, bear, and canids were made in layers deeper than previous recorded zooarchaeological analyses, extending the presence of these taxa at the site. The unexpectedly high level of collagen preservation in the bones from the cave confirms the potential for further applications of biomolecular approaches to Pleistocene bones from southern Italy.

**Supplementary Information:**

The online version contains supplementary material available at 10.1038/s41598-025-11355-6.

## Introduction

Biological and cultural changes that occurred during the spread of anatomically modern humans through Western Eurasia between 50,000 and 35,000 years ago is a continuing and challenging field of research, particularly in terms of the timing, interaction with, and replacement of Neanderthals. In this time span the archaeological scenario has yielded a multifaceted mosaic of cultural entities, exhibiting a variety of technological approaches, symbolic traditions, and subsistence strategies, reflecting a diverse range of behaviours when hominins interacted with their environment^1–8^.

Italy, and especially its southern part, was identified since the 1960s as a significant region in our understanding of the appearance of modern humans and disappearance of Neanderthals^9–11^. This is due to the occurrence of key archaeological sites with stratigraphic sequences that document the succession of the Late Mousterian, Uluzzian and Protoaurignacian/Early Aurignacian (e.g. Grotta della Cala, Grotta di Castelcivita and Grotta di Serra Cicora), which follow each other in stratigraphic order with no interstratifications. In addition, a stratigraphic hiatus is recorded between the late Mousterian occupation and the earliest Upper Palaeolithic layers at most sites^2,12–15^. Several southern Italian sites have provided significant information for the period, most notably the site of Grotta del Cavallo in Apulia with the earliest modern human remains from Italy^16^.

**Fig. 1:**
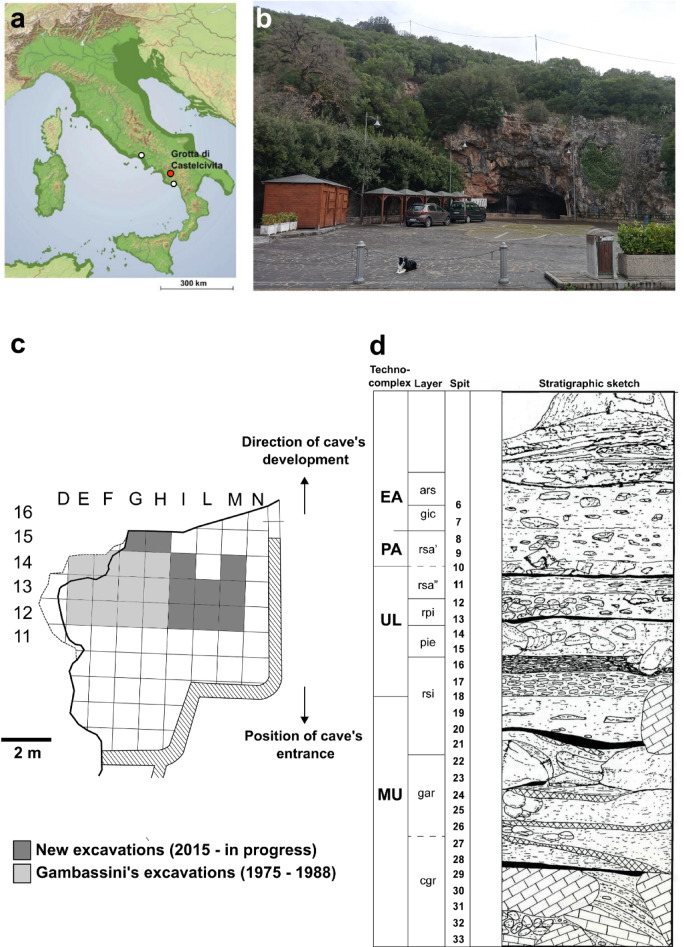
(**a**) Red dot showing location of Grotta di Castelcivita in southern Italy with white dots indicating neighbouring sites of Grotta della Cala (south of Castelcivita) and Grotta Roccia San Sebastiano (north of Castelcivita) with similar chronology, sea level 70 m below present-day (Benjamin et al. 2017) (**b**) the cave’s entrance (photo taken by Adriana Moroni) (**c**) excavated area (**d**) stratigraphic sequence of Grotta di Castelcivita. (modified from^17^). MU = Mousterian, UL = Uluzzian, PA = Protoaurignacian, EA = Early Aurignacian.

Grotta di Castelcivita, on the west side of the Italian Peninsula (Fig. 1a), has been considered of paramount importance, due to its high-resolution Palaeolithic sequence yielding evidence of Late Mousterian, Uluzzian, Protoaurignacian^17–19^ and Early Aurignacian occupations^20,21^. The anthropogenic sequence is sealed by a thick multilayered flowstone incorporating thin layers of pyroclastic materials identified as the byproducts of the Campanian Ignimbrite eruption (CI-Y5) that occurred at 39.85 ± 0.14 ka BP^21–23^ which constitutes a *terminus ante quem* for the Palaeolithic human presence at the cave. The site has been dated recently using radiocarbon and luminescence techniques, and a series of new determinations have helped illuminate its chronological sequence in great detail^6^. The Mousterian lithic assemblage (dated between 47,800-44,000 and 43,700–42,800 cal. BP 95% probability^6^) is made from local chert, fine-grained quartzarenite, radiolarite, and limestone, and is dominated by the unidirectional and convergent Levallois reduction strategies, aimed at producing standardised flakes and blades. Retouched tools are mainly sidescrapers, long sidescrapers, and points^17^ (Marciani, ongoing study). During the Uluzzian (dated approximately from 43,350-42,600 to 40,400–39,850 cal. BP^6^) the used lithotypes are still represented by local chert, quartzarenite, radiolarite, and limestone. Production usually began on unmodified natural surfaces using cores organised along parallel and orthogonal planes, as well as semi-tournant configurations. This procedure resulted in less standardised artefacts compared to Levallois or volumetric blade reductions. Bipolar technique is dominant, producing a broad range of flakes, flakelets, and bladelets in varying shapes, sizes, and thicknesses^18,19^. Among retouched tools, lunates and short end-scrapers are very typical. Bone tools and ornaments on shell are also present^17,20^. The Aurignacian is chronologically constrained between the end of the ULuzzian and the CI (39.85 ± 0.14 ka BP)^21–24^. Both in the Protoaurignacian and Early Aurignacian phases, locally sourced fine-grained chert was used. The production focused on the obtainment of bladelets from prismatic cores by direct percussion. The occurrence of long, straight Dufour-type bladelets is the distinctive feature of the Protoaurignacian^21^. In the Early Aurignacian bladelets were produced from carinated cores, with the smallest blanks being directly retouched to form pointed distal ends^21^. Bone tools and ornaments made from marine shells were also recovered^20^. Several hearths associated with short-term palimpsests were found in the Uluzzian and the Aurignacian layers^17^.

The Castelcivita assemblage spans an important period in which both Neanderthals and modern humans coexisted in Europe and new hominin fossils would be valuable additions to understanding human evolution and differences in adaptation and subsistence strategies of the different hunter-gatherer groups establishing themselves in new territories. Palaeoproteomic studies in Eurasia have revealed new hominin remains, as well as helped expand ranges and ages of known hominin species^3,25–30^. The aims of this study were threefold: (i) to establish whether collagen preservation in the Pleistocene-age bones from Castelcivita was sufficient for collagen-based analyses (palaeoproteomics, radiocarbon dating of bone); (ii) to apply Zooarchaeology by Mass Spectrometry (ZooMS) to unidentifiable bone fragments (> 2 cm) from Middle and Early Upper Palaeolithic contexts of the site as a way of complementing the traditional zooarchaeological dataset; and (iii) to identify new hominin remains from the undiagnostic fragmentary bones from the Castelcivita faunal assemblage. Our study represents the largest application of ZooMS on Palaeolithic material from the Italian peninsula spanning three important cultural phases: the Late Mousterian, Uluzzian, and Protoaurignacian. The former is traditionally linked to Neanderthals, whereas the other two can be associated with the earliest incoming modern human groups entering Europe^1,2,14^.

Following from the recent dating efforts at Castelcivita^6^ (where the oldest radiocarbon ages were obtained from the upper Mousterian layer *rsi*), here we apply palaeoproteomic techniques to fragmented bones excavated at the site to reveal new insights into hominin presence and the shifting ecological setting of the site by also targeting the deepest material from the Mousterian layers (*gar* and *cgr*). In addition to potential differences in the subsistence behaviours between indigenous Neanderthals and migrating modern humans, an understanding of the taxonomic diversity and quantity of fauna observed between these three cultural phases can be used to infer various climatic shifts. Hence, combining ZooMS with traditional taxonomic zooarchaeological data provides two possibilities: (1) the ZooMS results will mirror the zooarchaeology, or (2) differences in ZooMS data will reflect changes in climate/ecology that are not visible in the macrofauna record. The addition of ZooMS identified material is expected to not only increase the number of identified specimens but also increase the taxonomic diversity at the site, which may elucidate variations in faunal patterns over the three cultural complexes.

### Site and environmental background

Grotta di Castelcivita (40.495636, 15.209222) lies at the foot of the Alburni massif, in the province of Salerno with the archaeological deposit located at the mouth of the cave (Fig. 1a-b). Systematic excavations at the site by the University of Siena between 1975 and 1988 (dir. Prof. P. Gambassini) covered an area of over 14 m^2^^17^. New excavations from 2015 onwards (dir. A. Ronchitelli, A. Moroni and S. Arrighi) have expanded the original trench and revealed new material for scientific analyses (Fig. 1c). The site includes a 2.6 m deep rich anthropogenic sequence. The main sedimentological, stratigraphic, and archaeological description of layers reported^31^ is integrated with new data^21^. The main features from the bottom to the top of the succession are presented in Fig. 1d and below.

*Stratigraphy*.

- *Layer lower cgr (spits 33 − 29)*.

Late Mousterian. This layer forms the base of the succession at Castelcivita and consists of a thick level (about 2 m) of rock fall deposits consisting of large limestone blocks and debris, whose origin is related to collapse from the cave vault and walls. Fine-grained sediments are infiltrated within the blocks. This level possibly lies on the cave bedrock, which however has not been exposed.

- *Layers upper cgr and gar (spits 28 − 24)*.

Late Mousterian. These two layers directly overly layer lower cgr and consist of fine-grained sediments (silt and clay) with scattered debris and clasts, at places, cemented. The deposits locally incorporate pyroclastic materials. Large limestone blocks occur at top of layer gar.

- *Layer lower rsi (spits 23-lower 18)*.

Late Mousterian. The layer is formed by fine-grained sediments (clay, silt, and fine-grained sand) with scattered debris and a weak plane-parallel lamination. In places cemented sub-layers occur.

- *Layers upper-rsi*,* pie*,* rpi and rsa” (spits upper 18 − 11)*.

Uluzzian. These deposits are about 80 cm-thick, and consist of fine-grained materials (silt and clay), occasionally mixed with more coarse-grained materials like sand and granules, and common breakdown debris. These deposits attest to a change in sedimentation which becomes dominantly allochthonous with subordinated autochthonous sediments.

- *Layer rsa’ (spits 10–8)*.

Protoaurignacian with marginally backed bladelets (Dufour) (see Supplementary Information 4 for more detail on lithics at the site). Fine-grained reddish sediments dominated by silt and sand with scattered debris. Rounded clasts point to a completely allochthonous origin for these sediments. Layers rsa” and rsa’ are almost identical from a sedimentological point of view but differ in terms of their material culture.

- *Layer gic (spits upper 8–7)*.

Early Aurignacian with micro-points of the Castelcivita type. Yellowish fine-medium-grained sand, locally rich in a muddy matrix. Rounded clasts are common in the lower part, with some clasts made of poorly consolidated pyroclastic material. Deposits of this layer are slight to firmly cemented.

- *Layer ars (spit 6 − 4)*.

Early Aurignacian with micro-points of the Castelcivita type. Reddish to yellowish fine-grained sand with a silty matrix. Pyroclastic materials occur as small clasts within the bed.

*Chronology*.

A new chronological framework has been recently established for the site^6^ adding to previously published studies^24,32,33^(see Supplementary Information 4, Table S5 and S6 for an overview of the site chronology). The earliest Mousterian occupation of Castelcivita remains undated due to the lack of suitable material for radiocarbon dating, however, the Mousterian portion of layer *rsi* contains the oldest radiocarbon dates between ~ 41,700-37,800 BP. Whereas, spit 24 of layer *gar* show the earliest dated level from the site with an optically stimulated luminescence (OSL) age range between 47,800-44,000 cal BP (all ranges are given at 95% probability)^6^. The Mousterian seems to fall well within Greenland interstadial (GI) 12, a long warming period of Marine Isotope Stage 3. The latest Mousterian occupation in spit 19 of layer *rsi* is followed by a phase of abandonment of the cave by humans, dated to between 43,700-42,800 cal BP. The start of the Uluzzian, following a depositional gap marked by an erosive event, is also estimated to start at 43,350-42,600 cal BP, hence it is not possible to calculate confidently how long the absence of humans lasted. The end of the Uluzzian at the end of spit 11 of layer *rsa”*, is directly followed by the Protoaurignacian (layer *rsa’*). Several radiocarbon dates from this part of the sequence point to a relatively short phase dating to between 40,400 and 39,850 cal BP. The Early Aurignacian levels (*gic*, and *ars*) are ultimately capped by the volcanic deposits attributed to the Y5 (Campanian Ignimbrite) eruption dated 39.85 ± 0.14 ka BP^22,23^. These are embedded in a thick multilayered flowstone reaching the ceiling at the entrance of the cave and definitively sealing the archaeological sequence.

*Zooarchaeology*.

Previous zooarchaeological analyses were undertaken on bird and fish remains^34^ and on large mammals^35^. Recently, a comprehensive taphonomic study was conducted on bird specimens^36 ^along with a preliminary taphonomic analysis on macromammals^37 ^as well as a revision of the large mammal sample which is ongoing by some of the authors (FB and JC). Large mammal bones are present in the cave mainly due to humans^35 ^although the presence of hyaenas is attested throughout the sequence. The zooarchaeological analyses of the identifiable large mammal remains identified distinct differences between the three main periods (i.e. Mousterian, Uluzzian and Aurignacian) of the site. The larger percentages of cervids and the scarcity of horse during the older periods may be taken as evidence of higher humidity/precipitation in the region. Horses become more abundant during the Upper Palaeolithic together with large bovids. A trend towards more arid and colder climate is also attested by the analysis of birds and small mammals^35^.

The faunal patterns observed for Castelcivita can be compared directly with the other Campanian sites bearing Late Mousterian-Uluzzian-Aurignacian layers (see Fig. 1a for locations), such as Grotta della Cala and Grotta Roccia San Sebastiano^38^. Similar patterns regarding the presence of large mammals are observed for the Uluzzian layers of Castelcivita (layers *pie* and *rpi*) and the other two sites, characterised by an abundance of cervid taxa and a reduced presence of equids^39,40^. In the Aurignacian, both Cala and Castelcivita (layer *gic*) show a dominance of red deer accompanied by low percentages of other taxa, like horse, boar, other cervids, caprines, and large bovids.

## Results

As stated before, a revision of the whole large mammals zooarchaeological sample is in progress, and data collected so far are very preliminary and their distribution across layers is uneven. Nevertheless, on a sample of more than 20,000 remains (both taxonomically identified and unidentified), bones are not affected by erosional phenomena, weathering is rare (only 13 specimens in the whole sample) and carnivore-induced modifications are equally distributed across Late Mousterian, Uluzzian and Protoaurignacian (between 0.4 and 0.5% of remains). Anthropic marks are identified at least three times more than carnivore marks at the site (between 1.6 and 3.7% of remains). The anthropic origin of notches is still under evaluation, but are often associated with cutmarks, however, are never associated with tooth marks. Among diaphyseal fragments, those bearing green-bone fractures are the most abundant (284 on a sample of 303 specimens in the whole site).

Preservation of bone collagen at the site was unexpectedly high (compared to previous studies in southern Italy^41^) and the majority of bones were able to be identified using ZooMS. In total, 1114 out of 1263 bone fragments (Table 1) from Castelcivita were identified to genus or family using the ZooMS faunal categories (Supplementary Information 2). For complete ZooMS results see Supplementary Information 3. The majority of fragments (ZNISP = ZooMS Number of Identified Specimens) came from the Uluzzian layers (*n* = 840) (Table 1), which had an 87% identification success rate, whereas the least number of bone fragments came from the oldest Mousterian layers, however, these had the greatest preservation and a successful identification rate of 91%. To ensure comparability between the ZooMS and morphological NISP (Number of Identified Specimens) values (Table S1) the ZooMS categories were simplified into broad faunal categories (Table 1) that also covered the zooarchaeological morphology-based results^35,36^. NISP values were originally recorded for the morphologically identified samples in each layer, but interpretations regarding abundance, diversity, and fragmentation rates are limited based on the current quantification and diversity data available.

We did not identify any human remains using ZooMS, however, various faunal taxa were identified. *Equus* sp. was the most commonly identified group with 448 bones (Table 1), followed by cervid, possibly including giant deer (*Megaloceros giganteus)*, fallow deer (*Dama dama*), and red deer (*Cervus elaphus*), and then followed by caprines and *Bos*/*Bison*. The broad faunal category ‘Cervidae’, possibly including giant deer, roe deer (*Capreolus capreolus*), fallow deer, and red deer, contains 367 bones, which makes Cervidae the second most common faunal group identified through ZooMS. Caprines (including *Capra sp.*, *Rupicapra* sp. and unidentified Caprinae) are represented by 223 specimens. *Bos*/*Bison* follow, with nearly 145 identified specimens. Less common fauna at the site includes *rhinoceros* (*n* = 2) and carnivores, such as *Panthera*/*Crocuta*., canids, *Ursus* sp. and mustelids.

Variation in the faunal quantities occur between the three cultural techno-complexes (Mousterian, Uluzzian, and Protoaurignacian). Cervids dominate in raw ZNISP values in the Mousterian followed by caprines, whilst *Equus* sp. values are low (Table 1). Due to the lack of identifiable peptide markers (COL1a2 757) many of the unidentified cervids could not be further identified to either roe deer (*C*. *capreolus*) or red/fallow deer (*C. elaphus/D. dama*), therefore, the category ‘Unidentified Cervid’ includes all possible cervid species. While it is possible that greater numbers of roe deer were present in any of the three cultural-complexes, it appears that roe deer were more common in the Uluzzian. Despite equids dominating the faunal assemblage in the Uluzzian, overall cervidae numbers are also high along with an increase in *Bos*/*Bison* and chamois (*Rupicapra* sp.) in the Uluzzian compared to the Mousterian.

**Table 1:** ZooMS identified taxa with number (ZNISP) and NISP % for each cultural complex (Mousterian, uluzzian, and Protoaurignacian). ZooMS ID’s correspond with faunal categories that can be compared to the morphological NISP assemblage (see Table S1 for species-specific identifications). Morphological NISP also presented based on ZooMS ID.

Faunal categories	ZooMS ID	Mousterian			Uluzzian			Protoaurignacian (RSA’ only)			ZooMS Total	Morph Total
		ZNISP	ZNISP %	Morph NISP	ZNISP	ZNISP %	Morph NISP	ZNISP	ZNISP %	Morph NISP		
Equid	*Equus*	3	1.4%	3	338	40.2%	73	107	50.2%	10	448	86
Cervidae	Cervid	118	56.2%	144	107	12.7%	69	23	10.8%	7	248	340
Cervidae	*Capreolus capreolus*	1	0.5%	29	12	1.4%	34	0	0.0%	2	13	65
Artiodactyla	Cervid/Rupicapra	2	1.0%	-	0	0.0%	-	0	0.0%	-	2	-
Bos/Bison	*Bos*/*Bison*	13	6.2%	14	96	11.4%	17	47	22.1%	6	156	37
Caprine	*Capra* sp.	21	10.0%	53	14	1.7%	12	4	1.9%	1	39	66
Caprine	*Rupicapra*	8	3.8%	85	52	6.2%	25	5	2.3%	-	65	110
Rhinoceros	Rhinoceros	2	1.0%	1	0	0.0%	-	0	0.0%	-	2	1
Cervidae	Unidentified Cervid	15	7.1%	17	90	10.7%	5	1	0.5%	-	106	22
Caprine	Unidentified Caprine	1	0.5%	-	3	0.4%	-	2	0.9%	-	6	-
Artiodactyla	Artiodactyla	0	0.0%	-	1	0.1%	-	0	0.0%	-	1	-
*Sus* sp.	*Sus* sp.	2	1.0%	7	13	1.5%	8	0	0.0%	7	15	23
Carnivore	*Ursus* sp.	3	1.4%	1	2	0.2%	5	0	0.0%	-	5	6
Carnivore	Canid	1	0.5%	-	1	0.1%	5	0	0.0%	-	2	5
Carnivore	*Felis*/*Lynx*/*Ursus*	2	1.0%	-	0	0.0%	5	0	0.0%	-	2	5
Carnivore	*Panthera*/*Crocuta*	0	0.0%	15	0	0.0%	12	1	0.5%	-	1	27
Carnivore	Carnivora	0	0.0%	1	3	0.4%	7	0	0.0%	-	3	8
	Fail	18	8.6%	-	108	12.9%	-	23	10.8%	-	149	-
	**Total**	**210**		**481**	**840**		**287**	**213**		**33**	**1263**	**801**

**Fig. 2:**
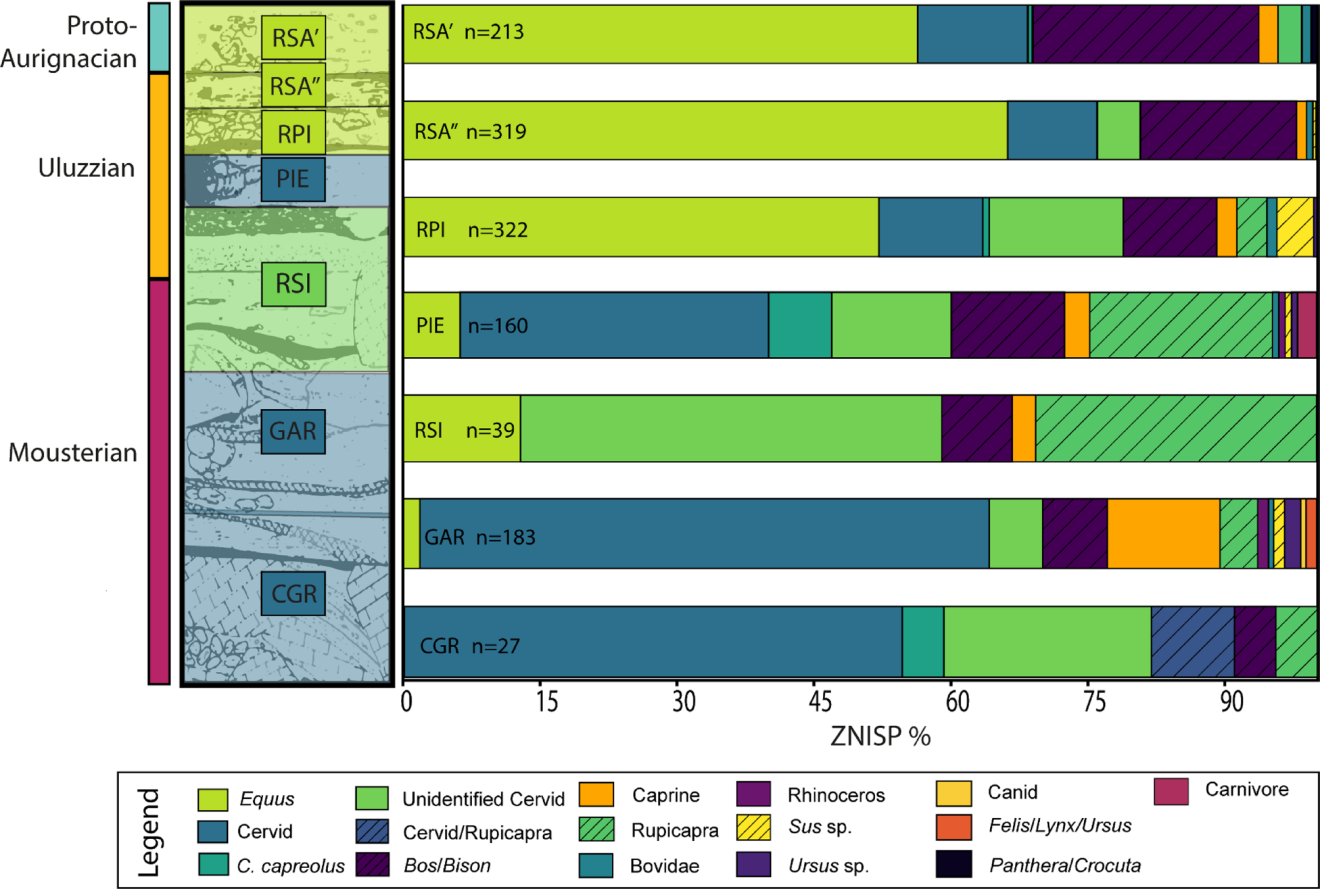
Abundance of ZooMS identified fauna at each stratigraphic layer represented as ZNISP percentage with the overall number of ZNISP presented as ‘n’. Note that the faunal colours are sequential in the legend top to bottom, left to right. The corresponding stratigraphy has been highlighted in the colour of the most abundant fauna for that layer (e.g. RSA’ in yellow corresponds with Equus). Samples from RSI are only from the Uluzzian layers. On the far left, a three colour scheme denotes each of the cultural complexes.

The clearest difference in dominant taxa shifting from cervids to equids is seen when separating the data into stratigraphic layers (Fig. 2). There is a clear change from cervids dominating in *cgr* and *gar* then decreasing and being replaced by equids and rupicapra in *rsi* and *pie*. *Rupicapra*, along with cervids, decrease and are heavily overtaken by equids in *rpi*, *rsa’*, and *rsa’’*. The highest diversity of taxa (primarily carnivores) is found in *gar* and *pie* layers, with both containing *Ursus* sp. and canid fragments not recorded in the morphological record, thus enlarging the taxonomic spectrum in these layers. On the other hand, the lowest diversity is seen in *rsi* and may attest to the semi-destructive protocol being used on all of these bone fragments^42^ and/or low sample numbers (see methods for more details). The morphological data from *rsi* (see Table S3) show similar species diversity in the surrounding layers, however no carnivores were identified with ZooMS.

### Comparing ZooMS and morphological determinations

The morphologically identified macrofauna from Castelcivita^35^ (Table S3) were placed into broad faunal categories which enabled comparison with the ZooMS identified results (see Table S2). For many of the categories and cultural complexes, ZooMS NISP values are greater than the original morphological NISP (Table S2). This is not the case for several of the Mousterian faunal categories due to the number of ZooMS samples in this period being substantially smaller than the morphologically identified material. Conversely, the total ZooMS samples from both the Uluzzian and Protoaurignacian are double the amount identified morphologically. Due to the nature of ZooMS sampling and high fragmentation of bone, ZooMS-identified values are greater than the morphologically-identified values and present more of the bone material in nearly all faunal categories. However, these identifications present an incomplete overview of each cultural complex due to the nature of ZooMS specimen selection of > 2 cm bone fragments and therefore only represent a portion of the overall assemblage. Additional ZooMS analyses on smaller bone fragments would provide a more complete indication of the macro and micro faunal assemblage and enable greater integration of the ZooMS and morphological datasets. Nevertheless, a number of observations can be made with this additional data. Whether fragmentation is greater in some taxon rather than others may be a key variable in these NISP values, however, further analyses of specimen dimensions and taphonomy would be necessary to explore this in more detail. For example, larger bodied equids (estimated ~ 300–400 kg by some studies^43^) contain greater bone mass than, for example, caprine and can be assumed to create a greater number of unidentified fragments larger than 2 cm. Although measuring the thickness of the diaphysis or estimating its circumference can be used to distinguish animals of different sizes^44^ body size assignments^45,46^ are not always reliable and in our case the high fragmentation of the bones and the broad categories used reduce the usefulness of this method at Castelcivita. Nevertheless, various butchering processes and post-depositional factors can affect fragmentation counts and body size alone is not enough to understand the variation in NISP values.

**Fig. 3:**
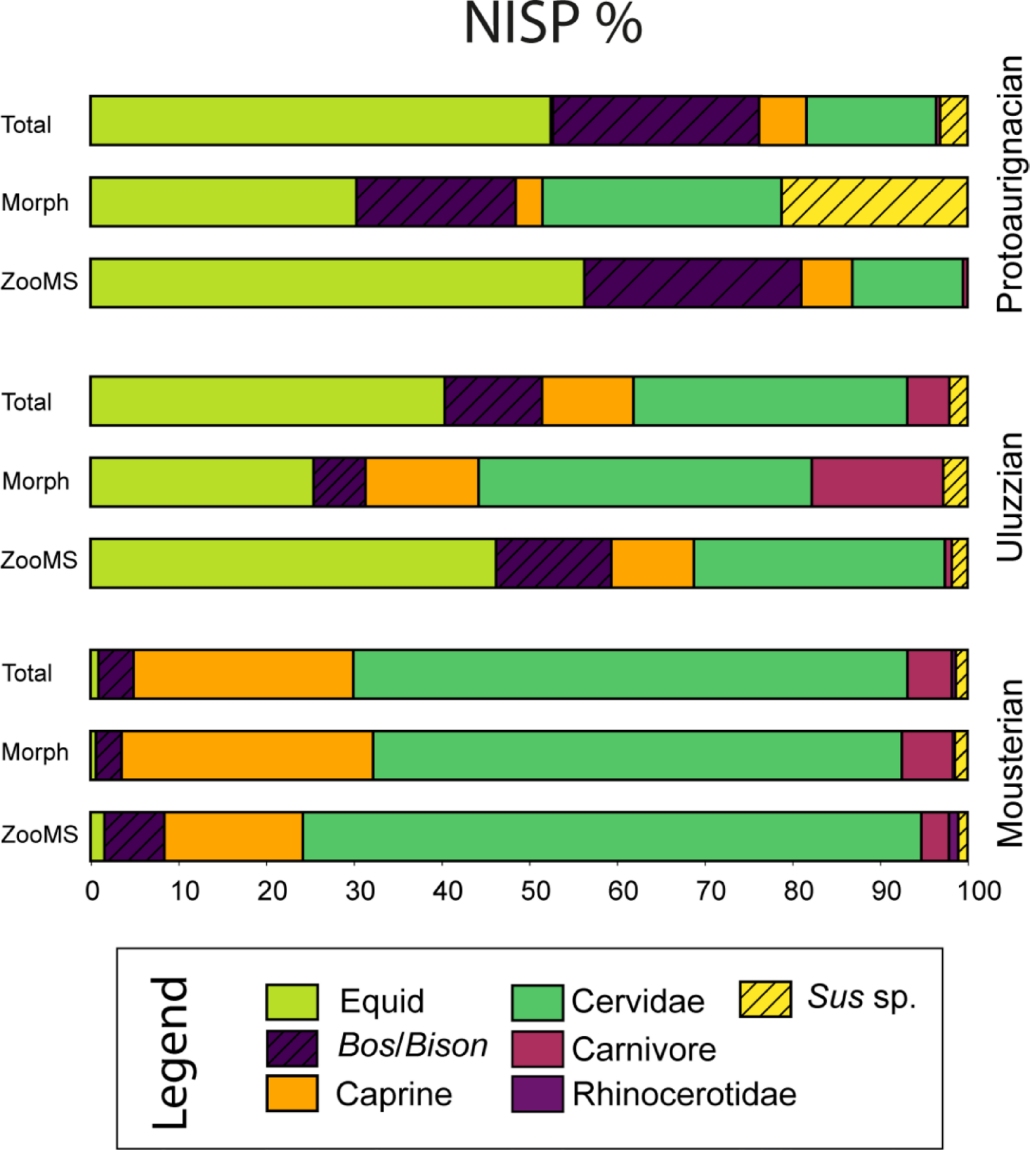
NISP values for ZooMS ID, Morph ID, and Total identified. Represented in broad faunal categories for comparability between the three cultural complexes. Protoaurignacian only includes RSA’ samples from Morph Ids as ZooMS was only sampled from this PA level. Artiodactyla values excluded as they are not present in the Morph Ids and therefore cannot be compared (See Table *S2* for raw values).

Figure 3 shows the NISP percentage (%) values for ZooMS ID, Morphological ID, and Total NISP for each of the categories based on cultural assignation. The Early Aurignacian layers (*gic* and *ars)* were not sampled for ZooMS so only the morphological material from the *rsa’* (Protoaurignacian) was used for comparison. 

Comparing any patterns in abundance and shift in faunal categories during the different cultural complexes between ZooMS and morphology are difficult due to the varied nature of these datasets. Instead combining the values into a total identified value provides a greater understanding of the faunal assemblage. Figure 3 shows the increased total NISP % values for combined ZooMS-identified and morphologically-identified material. Looking at these total NISP % values we see that overall abundance remains similar between the different methods - cervids are dominant in the Mousterian whilst equids dominate in both the Uluzzian and Protoaurignacian periods. Minor variations occur in the Uluzzian (identification of more equids and less carnivores) and Protoaurignacian (identification of more carnivores and less *Sus* sp.) between the ZooMS and morphological values that impact the total NISP % of each faunal category, however, patterns overall remain consistent. Statistical tests (Supplementary Information 4, Figure S1) also show that there is an association between the ZooMS and Morph NISP values, and linear bivariate model show a positive correlation between ZooMS and Morph values in each of the three cultural complexes.

### Collagen and spatial preservation

**Fig. 4:**
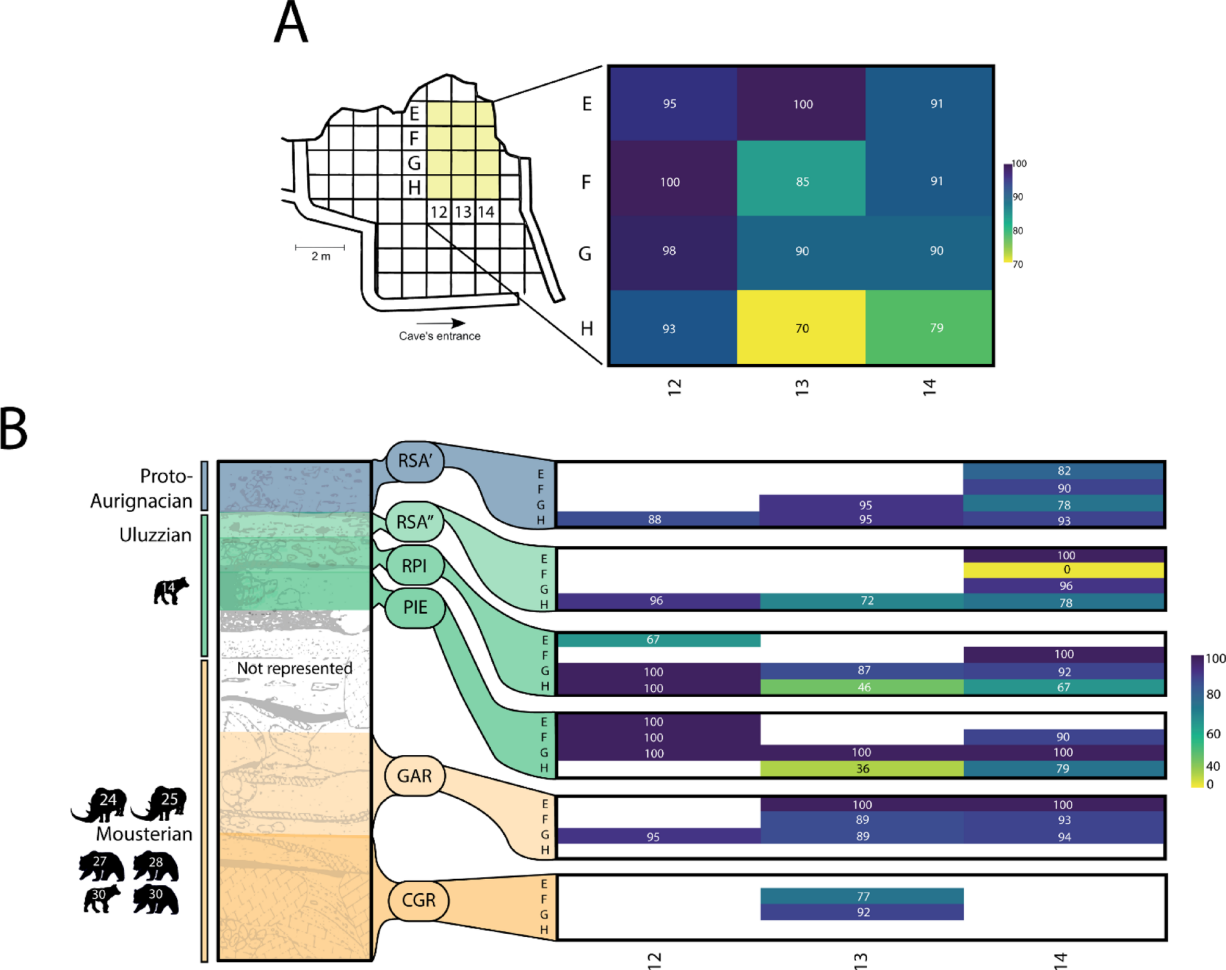
Successful ZooMS percentages separated spatially. **A** Percentage of successful ZooMS identifications in each square. **B** Successful ZooMS percentage of each square separated into levels (with x and y labels), from oldest (CGR) to youngest (RSA’) with gradient skewed to higher percentage as majority of values are between 80–100%. Corresponding strtigraphic profile and wider cultural layers shown with faunal silhouettes (phylopic.org) showing presence of specific taxa at depths (spit number in white) not previously recorded.

We wanted to identify whether collagen preservation was linked to a specific area of the site. Spatial data (square designations) was only available for 968 samples (analysed with acid insoluble protocol) and is presented as percentages of successful ZooMS identifications (samples with at least four peptide markers identified) in Fig. 4. The taxonomic identification of samples indicates the presence of collagen, however, various factors such as peptide extraction, enzymatic digestion, or mass spectrometry settings can also affect the degree of identifiable peptides and thus successful ZooMS results. This spatial comparison of results provides a simple overview of preservation at the site and can be further expanded with future studies (e.g. % N and deamidation) as well as including more detailed taphonomic analyses.

ZooMS identification rate was high throughout the 12 sampled squares and ranged between 70–100% (Fig. 4a). In Fig. 4a, the squares on the x axis (numbered) of “12” had the greatest success rate along with the y axis squares (letters) of “E”. When separating these results out by levels, variation in the percentage values diverge due to no square having material sampled from every level (Fig. 4b). For example, square F12 only had material from level *pie*, whilst F14 had every level apart from *cgr*. Figure 4b shows that squares G12, G13, G14 and H12 had high success rates over multiple levels (≥ 95%). The *pie* level has the most squares (5) with 100% identification rate, however it also has two squares with some of the lowest success rates. These are the squares H13 and H14, which have low percentages (36–79%) through *rsa”*, *rpi*, and *pie* levels (Uluzzian). These levels and squares contain some of the greatest numbers of sampled bone and are primarily dominated with *Equus* sp. and *Bos*/*Bison* ZooMS identified bones (Table S4). Despite having the most squares with the “lowest” success rates, the Uluzzian levels have some of the best preservation (Fig. 4b). Collagen preservation in the Protoaurignacian is fairly consistent and high in each of the sampled squares. This is also the case in the Mousterian levels, especially in *gar*.

An additional indicator of collagen preservation is presented in Supplementary Table S7 and S8, which demonstrate that the majority of successful taxonomic identifications are made using nine markers out of the maximum possible number of markers (9), throughout the stratigraphic layers of the site. Stratigraphic layer *cgr* had the lowest percentage for nine markers, with the majority of samples identified using between seven and nine markers.

### Newly identified taxa

ZooMS led to the identification of several taxa never previously attested in certain cultural complexes of the site (Fig. 4b). New rhinoceros bones were identified from the Middle Palaeolithic level *gar* (one from spit 24 (CLC476), and one from spit 25 (CLC503). The earliest and only evidence of rhinoceros at the site was a single morphologically identified bone (*Stephanorhinus* sp.) from Spit 21–22 in layer *gar*^35^. Given the stratigraphic age of these spits, these two new rhinoceros bones may be *Stephanorhinus* sp. or *Coelodonta antiquitatis*, however, further aDNA studies would be needed to distinguish between the two species. Additionally, new evidence of *Ursus* sp. in *gar* (*n* = 3) from spits 30 (CLC534), 28 (CLC400), and 27–28 (CLC372) was found using ZooMS. The earliest morphologically identified *Ursus arctos* and *Ursus spelaeus* was in the late Mousterian layer of *rsi*^35^. ZooMS samples now show *Ursus* sp. was present throughout the Mousterian, from Spit 30. Finally, two new bones of canid were identified in *gar* (Mousterian) spit 30 (CLC536) and in *pie* (Uluzzian) spit 14 (CLC43). Morphologically identified *Canis lupus* remains were only identified in the Protoaurignacian level^35^. Many of the same ZooMS peptide markers are found in Canidae species *Canis familiaris*/*latrans*/*lupus*, *Cuon alpinus*, and *Vulpes lagopus*/*vulpes*^30,47–49^. To differentiate between *Canis* and *V. lagopus*^49^ the COL1a2 889–906 peptide marker 1576.8 m/z is present in *Canis* and not in *V. lagopus*. Currently it is not possible to distinguish between *Canis* and *Cuon* species, therefore the ZooMS identification of ‘Canid’ was given (see Supplementary Information 2). These new ZooMS samples, along with the morphological identified material, suggest that canids were present at the site in each of the cultural layers. Overall, the new ZooMS-identified material extends the presence of rhinoceros, ursids, and canids at the site to even older deposits than previously recorded. Previous chronological studies^6,21^ and clear stratigraphy^17^ of the layers, indicate that these bone fragments did not move through the stratigraphy, especially over the three cultural layers.

## Discussion

Although the traditional zooarchaeological record from Palaeolithic sites can be used as a proxy to infer palaeoclimate^50^ it cannot be considered (like plant macro-remains) as representative *in toto* of the surrounding ecosystem, since taxa distribution is the result of a more or less intentional selection by hunter-gatherers. Additional isotopic analyses on bone or teeth, as well as pollen and charcoal analyses are also needed for robust palaeoenvironmental interpretations. The contribution of as much ecological data as possible is therefore essential for a reliable environmental reconstruction. In the case of Castelcivita^31,34–36^ the palaeoenvironmental reconstruction is based on the integration of several proxies (sedimentological, palaeobotanical, small mammals, macro mammals and birds), that provide a coherent picture of the climatic and palaeoenvironmental fluctuations that occurred at the site. In the initial Mousterian occupation (layers *cgr-lower gar*) the sedimentological data indicate a reduced morphogenesis and an abundance of vegetation also confirmed by charcoal analysis, which documents the presence of a forest composed mainly of mesophilus species, especially white fir^17^. The same conditions are evidenced by the faunal association with the abundance of cervid taxa (especially fallow deer, *Dama dama)* and numerous woodland forms (i.e. dormouse and hazel mouse) among the small mammals^34–36^. Bird remains (especially small Galliformes, such as the Grey and Rock Partridge species, as well as the Common Quail)^36^ also suggest a temperate climate. During the upper Mousterian (layers upper *gar*-lower *rsi*), the environment remains forested, but there is a change in plant taxa which include hazelnut, oak and Mediterranean pine. The association of large mammals still suggests a humid climate, but with an incipient cooling combined with a slight shift towards more open environments. The same signal could also be indicated by the marked increase in *Microtus* and by the abundance of rocky habitat species among birds. A distinct shift is seen during the Uluzzian period (layers upper *rsi-rsa”*) from the early stages, with colder and drier conditions, also identified by an increase in horse (*Equus ferus*) and common vole (*Microtus arvalis*), and clearly attested by sediments no longer autochthonous but transported from outside and by the tree cover composed more and more of heliophilous taxa^17,35^. These cold and arid conditions persist until the end of the Protoaurignacian (layer *rsa’*), which represents the peak of aridity. In layer *gic* (Early Aurignacian), the harsh climate of the previous phase is replaced by more temperate conditions mainly documented by the sedimentological record and the notable decrease of the horse, which is replaced by the red deer (*Cervus elaphus*) and the chamois (*Rupicapra* sp.)^35^. The thermal index of the birds, proposed in the already published taxonomic study^34 ^indicates a more temperate climate. The following Early Aurignacian layer (*ars*) represents the last brief cold episode mainly attested by the occurrence of arboreal taxa such as *Pinus* cf. *sylvestris/nigra and Betula*. Based on the study of charcoals, birds and micromammals, a thermal regime characterised by average temperatures lower than today’s seems to be detectable throughout the archaeological sequence.

There is no other evidence of such comprehensive palaeoenvironmental studies in Campania, also due to the extreme scarcity of sites documenting the transition from the Middle to the Upper Palaeolithic. Apart from Castelcivita, only the deposits of Grotta della Cala (Camerota - SA) and Grotta Roccia San Sebastiano (Mondragone - CE) contain the Mousterian-Uluzzian-Aurignacian sequence^2,6,38^. At both sites only the faunal remains have been published^39,41^ but it has to be highlighted, that zooarchaeological data are not available for the Mousterian of Grotta della Cala^37,39^ and some mixing was identified inside the Uluzzian layer of Grotta Roccia San Sebastiano^38^. Therefore, it is not currently possible to provide a reliable regional context and/or comparison for the period in question. Regarding the absence of faunas typical of cold and arid climates at Castelcivita, it should be noted that southern Italy was never inhabited by animals such reindeer, and there is no reliable evidence of the presence of other “cold taxa”, such as woolly rhinoceros during MIS3, as this area played a crucial role as a biogeographical hotspot influenced significantly by the Mediterranean climate^51^. This environment acted as a refugia for humans, flora and fauna in terms of geographical isolation, population survival, and species richness, thus continuously hosting temperate climate faunas. Nevertheless, shifts toward arid and continental conditions can be detected by the oscillations in the proportion of horse and caprine remains among large mammals^51^.

The taxonomic identifications using ZooMS were consistent to the morphologically identified faunal remains at the site and are in line with the fauna present in the region as well as at the time of site occupation. Faunal patterns remained relatively similar in each of the cultural layers with an initial dominance of cervids (excluding roe deer) and caprines in the Mousterian, which shifted in the Uluzzian with greater numbers of *Equus*, and *Bos*/*Bison* along with a decrease in overall cervids (apart from *C. capreolus* which was specifically identified only in the Uluzzian using ZooMS). This decrease in ZNISP for cervids and caprines continues into the Protoaurignacian (Fig. 2) along with an overall decrease in ZNISP. These NISP values may correspond with changes in the number of individuals and/or variations in fragmentation. As only the Protoaurignacian (*rsa’*) was sampled for ZooMS, the change of red deer and chamois replacing horse in the Early Aurignacian could not be compared or investigated^35^. Due to most ZooMS identifications being to genus or family level, examining and comparing nuances in species seen through morphological identifications in the sequence was not possible. Nevertheless, ZooMS taxon and categories show a similar pattern of environmental shift through time with species related to woody environments dominating in the Mousterian then shifting in the early Uluzzian, with an increase in horse related to colder and drier conditions and more open environments.

Variation between layers can be seen with changes in the dominant taxa in the ZooMS data (Fig. 2). The dominant taxa of cervids carry through the Mousterian into the early Uluzzian until layer *rpi*, where *Equus* dominates into the Protoaurignacian. Due to the lack of key peptide markers in our data to distinguish taxa between cervid, *Equus* and *Rupicapra*, it is possible that NISP percentages of cervid and *Equus* skew differently in reality. Nevertheless, current ZooMS identifications provide a good indication of faunal abundance and are in line with the morphological results^35^.

A complex record of collagen preservation is seen by the percentage of successful ZooMS samples (Fig. 4) throughout the sequence as well as the high degree of peptide preservation evidenced by marker counts (Table S7 and S8). The majority of successful samples contained nine out of nine markers and could be taxonomically identified to genus level. Even though material from a number of squares were not sampled, an overarching understanding of protein preservation for medium-large bodied fauna can be explored. In the majority of squares at least 80% of the sampled bone fragments preserved collagen and the site overall had 88% success rate (1263 ZooMS samples). The squares with the lowest percentages (H13 and H14), in the Uluzzian, primarily contain *Equus* sp. and *Bos*/*Bison* remains. This may indicate a taphonomic bias in the fragmentation and preservation of larger bodied equids and bovids in these squares/levels, perhaps due to differing processing, disturbance, or site-use aspects, however, further taphonomic analyses as well as deamidation analyses need to be undertaken to explore these possibilities. Alternatively, the failed samples may belong to other taxa that underwent processes impacting protein preservation. These data indicate that there is likely variability in taphonomic and post-depositional processes, spatially and temporally. Compared to other ZooMS sites in southern Italy where percentages of Uluzzian material were 76% at Roccia San Sebastiano and 89% at Uluzzo C^41^ Castelcivita has a high percentage of preserved collagen (81% for the Uluzzian), especially when considering the taxonomic determination of much of the samples to genus and species level. With such good preservation from the macro-faunal remains, the site holds much potential for future biomolecular research (e.g. radiocarbon dating, stable isotopes, and potential for aDNA), however, protein preservation assessed only by ZooMS analysis does not always imply the potential for radiocarbon dating or DNA recovery. Additionally, a combined examination of the faunal remains with taphonomic studies and collagen preservation may provide insights into depositional processes and time-averaging. This aspect has elucidated hunting strategies and the exploitation of various taxa at other Italian sites^41,45,52^ and is currently being explored.

**Fig. 5:**
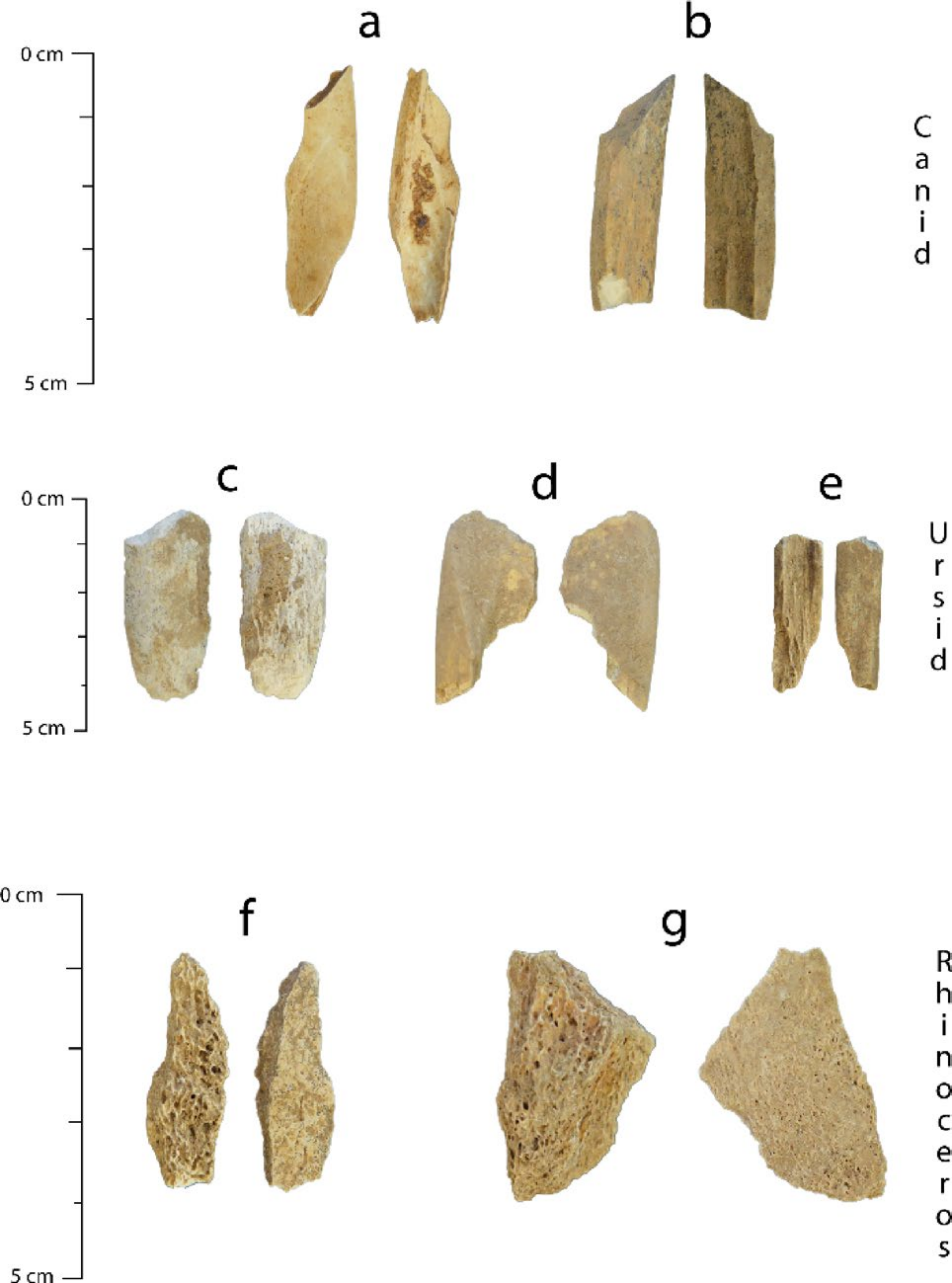
Newly identified taxa from originally unidentified fragmented bones. (**a**) CLC0043 Canid from spit 14, (**b**) CLC536 Canid from spit 30, (**c**) CLC372 Ursid from Spit 27, (**d**) CLC400 Ursid from spit 28, (**e**) CLC534 Ursid from spit 30, (**f**) CLC476 Rhino from spit 24, (**g**) CLC503 Rhino from spit 25.

A number of the ZooMS-identified samples were of taxa that were initially (morphologically) recorded in younger levels at Castelcivita^35^. New canids, *Ursus* sp. and rhinoceros bone fragments (Fig. 5) found (through ZooMS) in older deposits extend the period that Neanderthals or other predators (including hyaenas) were hunting or scavenging these taxa. Preliminary analyses of surface modifications on the bone assemblage suggests minimal carnivore-induced marks and more likely indicates these newly identified fragments are linked to human subsistence. The increase in NTAXA (number of taxa identified) in particular layers is expected due to the increase in NISP and has been recognised in other studies^53,54^.

Canid bones from the Uluzzian and Mousterian extend the presence of canids at Castelcivita significantly back in time. Both *Canis lupus* and *Cuon alpinus* were present in the region during the Late Pleistocene^55–57^ and *Canis lupus* (NISP = 3) was identified morphologically at Castelcivita in Protoaurignacian spits^35^. Therefore, it is possible the two canid specimens could be either species. Whether these remains were purposefully brought into the cave by Neanderthals (Mousterian) or early *Homo sapiens* (Uluzzian), or died there naturally, is not clear from the fragmented bones alone. Masini and Abbazzi^35^ argue that carnivore presence at the site was a result of human hunting, as there is an absence of articulated skeletal elements in the morphologically identified faunal assemblage. This seems likely with the *pie* canid bone fragment (CLC43), however, the *cgr* bone (CLC536) comes from a Mousterian layer with few morphologically identified carnivore remains. Therefore, it is less clear based on current evidence whether the absence of connected elements is due to hunting or high fragmentation and weathering of the material and taphonomic processes. Carnivore ZNISP and NISP % values are the highest in the Mousterian (Table 1; Fig. 2) and may indicate occasional visitation/denning or alternatively a more intensive hunting of carnivores than previously understood.

If canids were brought into the cave by hominins, the question arises whether this was as prey/ritualistic objects or as commensal animals. Current evidence shows that the first synanthropic wolves or palaeo-dogs occurred ~ 30,000 years ago^58–61^ and the first genetic dogs approximately 15,000 years ago^62–67^. There has been much debate and discussion on the domestication of wolves^64,68–72^ and the presence of Late Pleistocene dogs in Italy^73,74^as well as evidence of cut-marked wolf bones from Initial Upper Palaeolithic (IUP) sites like Bacho Kiro Cave and Ranis that suggest targeted resource use^75^. However, further dating, genetic, and isotopic work needs to be done on these canid samples from Castelcivita to better understand their chronology and relationship to hominins.

The earliest morphological evidence of *Ursus* sp. at Castelcivita was recorded in the late Mousterian (lower *rsi*), however, ZooMS shows that *Ursus* sp. was present even earlier in *cgr* (spit 30) and maybe even in spit 31 (in the group of *Felis*/*Lynx*/*Ursus*). The small numbers of ursid remains at Castelcivita suggest bear remains were brought in by predators/scavengers rather than the cave used as a ‘bear site’^76^. Numerous Middle Palaeolithic European sites exhibit evidence of bear exploitation^77,78^ with Neanderthals interacting and targeting bears for fur, meat, and bones. Examination of these *Ursus* sp. bones showed no evidence of cut marks, knapping, or burning, and no distinct ZooMS markers are currently known that could distinguish between Cave or brown bear, or *Ursus* and *Felis*/*Lynx*^79^.

The newly identified rhinoceros from the Mousterian level *gar*, match with a previous identification of a Rhinocerotin indet. fossil from the same *gar* level^35^ however, the two newly identified bones come from deeper deposits (spits 24 and 25). Although ZooMS cannot currently differentiate between different rhinoceros species, these bone fragments are likely to be either of the megafaunal rhinoceros (*Stephanorhinus hemitoechus* or *Coelodonta antiquatatis*), which became extinct in Italy around 41 ka BP^80,81^ These results also confirm the presence of rhinoceros only in layer *gar*. Previous chronological work^6^ date layer *gar* to 48.6 ± 3.3 ka (OSL), which suggests these specimens to be of a similar age. In addition, oxygen isotope records^82^ indicate this period contains a partial overlap of relatively cold conditions that would have suited megafaunal rhinoceros, as well as morphologically identified *Ursus spelaeus*^35^ (which may include the ZooMS identified *Ursus* sp. samples).

Regarding evidence for megafauna, no new *Megaloceros giganteus* bones were identified, however, as the peptide markers for this species^30^ are the same as fallow and red deer it is possible that some ZooMS identified cervid/unidentified cervid samples are in fact *M. giganteus*.

## Conclusion

Our study highlighted the successful application of biomolecular methods on Late Pleistocene material from an assemblage in southern Italy with a significant record of Neanderthal and *Homo sapiens* presence. ZooMS analyses on 1263 bone fragments from various levels throughout the Mousterian, Uluzzian, and Protoaurignacian revealed several new insights. We observed a surprisingly high ZooMS identification rate with 88% of all the sampled bone able to be identified, the majority of which to genus or species. These additional ZooMS results increased the overall NISP and mirrored the zooarchaeological record. Furthermore, similar taxonomic patterns are seen between the original morphometric zooarchaeological analyses and the ZooMS results, with the dominant taxa changing due to climate shifting from woody temperate and humid environments to more open landscapes with colder and drier conditions. Nevertheless, greater taxonomic diversity was seen in a number of layers with the addition of ZooMS. Finally, new ZooMS identifications of canid, *Ursus* sp. and rhinoceros in older (Mousterian) deposits than previously recorded expand the record of these taxa at the site and in the region. Despite no hominin remains being identified at the site, this is the largest application of ZooMS in Italy and the results highlight the exciting possibilities for biomolecular approaches in these Late Pleistocene European assemblages, previously considered devoid of biomolecular preservation and hence not analysed.

### Methods

This study was undertaken in two stages. An initial pilot study focused primarily on material from the Uluzzian layers (*rsa’’*, *rpi*, *pie*, *rsi’*) and targeted 295 unidentified bone fragments (Bell, 2018 MSc unpublished thesis^42^). The samples were taken from a collection of unidentified fragmentary bones (no distinguishing morphological features present to identify age or type of bone) from the 1976 and 1982 excavations at Castelcivita. They were selected on the basis of their size (> 2 cm in length or width). However, spit “lower 16” was underrepresented due to a lack of suitable bone fragments. Each bone was drilled with a tungsten carbide drill to produce a bone powder of 20–50 mg at University of Siena (Italy).

The second stage of this study was focused on expanding to more layers with an additional 968 samples (> 2 cm in length) from the Mousterian, Uluzzian and Protoaurignacian layers from Gambassini’s 1975–1988 excavations. Bone chips were drilled using a 2.35 mm drill and equipment and surfaces were cleaned with ethanol and sandblasted between samples. In total 1263 samples were analysed using ZooMS, along with blanks/control tubes added for every 24 samples.

The semi-invasive ammonium bicarbonate (AmBic) protocol^47,83^ was used for the initial 295 samples, whereas the destructive acid insoluble protocol^47,84^ was used for the remaining 968 samples as this protocol has a higher success rate. The main differences between these two protocols concern the initial steps of the process, either using buffer extraction (semi-destructive) or HCl-based demineralisation (destructive). For the AmBic protocol, the sample is immersed in 100 µl of 50 mM AmBic solution in MilliQ water and is left overnight at ambient temperature. The supernatant is then discarded and another 100 µl of AmBic solution is added prior to gelatinization.

For the acid insoluble protocol, 500 µl of cold 0.6 M HCl is added to each sample and left in the fridge at 4 °C until the bone chip becomes flexible/spongy and no bubbling is visible. This took approximately 3–5 days for the 968 Castelcivita samples. Once demineralized the samples were centrifuged and the acid supernatant removed. The remaining bone was then washed using 200 µl of 50 mM AmBic solution, briefly mixed, centrifuged and then the solution was discarded. This process was repeated 3 times. Finally, 100 µl of AmBic was added to each sample for gelatinisation.

All 1263 samples were placed in an oven at 65 °C for 1 hr to help release further collagen from the bone matrix into the AmBic solution, and denature it into gelatin. Samples were then centrifuged and 50 µl of extract was removed for digestion. 1 µl of trypsin (0.4 µl/µg Pierce™ Trypsin Protease, Thermo Scientific) was added to the extract and the sample was incubated overnight (12–18 h) at 37 °C. After centrifuging, 1 µl of 5% TFA (trifluoracetic acid) was added to stop the trypsin and the extract was desalted and purified using C18 Ziptip (Pierce™ C18 Tips, Thermo Scientific) into 50 µl of conditioning solution (for the 968 samples). For the 295 samples extracted at the University of Oxford, after adding the 5% TFA to stop the trypsin, 96-well-plate insolute solid phase extraction (SPE) cartridges (215 mg; Biotage, Uppsala) were prepared with 1 ml of washing solution (0.1% TFA in MilliQ water). Once this finished draining, 1 ml of conditioning solution (0.1% TFA in 50:50 Acetonitrile and MilliQ water) was pipetted into each cartridge. 50 µl of the sample was then added into the corresponding cartridges and then 200 µl of conditioning solution was added to extract the hydrophobic peptides into an empty 96-well plate below. The solution was then evaporated for transportation to the MALDI-TOF mass spectrometer at the Max Planck for Geoanthropology in Jena, Germany. The samples were solubilized using 100 µl of conditioning solution.

### Spotting, MALDI-TOF analyses and collagen mass fingerprint identification

Next, 0.5 µl of each sample was spotted on a target MALDI plate (MTP ground steel) in triplicate (Supplementary Information 1), and 0.5 µl of matrix solution (10 mg of d-cyano-4-hydroxycinnamic acid in 1 mL of conditioning solution) was added on top of each sample, and were left to crystallise and dry. Calibrant spots were also added on the plates as they have known peptide sequences which ensure consistency between samples and help identify instrument drifts. Once dry, the plates were run through a Bruker AutoFlex LRF Speed MALDI-TOF-MS (Bruker Daltonics) with AutoXecute run on reflector mode, with laser power fuzzy control ‘on’, initial laser power = 55, maximum laser power = 90, random walk, and sum of shots = 800. The files were exported and analysed in mMass (v 5.5.0^85^). The spectra obtained by the MALDI-TOF-MS were compared to publicly available markers and a user-informed reference library (see Supplementary Information 2 and 4) after initial baseline correction (Precision = 15, Relative offset = 25), smoothing (Method = Savitzky-Golay, Window size = 0.3, Cycles = 2), and peak picking (S/*N* = 3.0, Picking height = 75%) in mMass. There were no issues of contamination visible in the blank controls. Spectra triplicates were identified individually and checked to ensure matching markers and then were combined.

Taxonomic category divisions (Supplementary Information 2) were made based on similar markers present in varying species. For example, for peptide marker COL1ɑ2 757–789 (G) the m/z values 3017/3033 distinguishes Cervid from *Capreolus capreolus* with *m/z* at 3043/3059. The absence of certain markers in the spectra results in additional species as the possible taxonomic identification, which broadens the category (e.g. peptide marker COL1ɑ2 502–519 (C) is not present, therefore, *m/z* 1580 or 1550 cannot be used to confine the possible species any further than Cervid and *Rupicapra rupicapra*).

## Electronic supplementary material

Below is the link to the electronic supplementary material.

Supplementary Material 1

Supplementary Material 2

Supplementary Material 3

Supplementary Material 4

## Data Availability

All data generated or analysed during this study are included in this published article [and its supplementary information files]. The datasets generated and/or analysed during the current study are available in the Zenodo repository, https://doi.org/10.5281/zenodo.14068163.
