# Supplementary material for: New insights from the application of ZooMS to Late Pleistocene fauna from Grotta di Castelcivita, southern Italy
Source: Sci Rep. 2025 Jul 17;15:25906. doi: 10.1038/s41598-025-11355-6 (PMC12267457; doi:10.1038/s41598-025-11355-6)
Supplement: Supplementary file 4 — Supplementary Material 4 [file 41598_2025_11355_MOESM4_ESM.docx]

**Supplemental Information 1**: MALDI plate maps (see excel spreadsheet)

**Supplemental Information 2**: ZooMS faunal categories with peptide markers (see excel spreadsheet for selected taxa)

**Supplemental Information 3**: All samples with ZooMS peptide peaks identified and taxonomic identifications made.

(see excel spreadsheet)

An online database of published ZooMS peptide markers can be accessed through the University of York.

https://www.york.ac.uk/archaeology/research/research-centres/bioarch/research-themes/palaeoproteomics/

Files corresponding to S1: Raw MALDI-TOF files (mzMl) found on Zenodo repository, https://doi.org/10.5281/zenodo.14068163

**Supplemental Information 4**

**Additional background on lithics**

In the Mousterian assemblage, the primary raw materials used for stone tool production included chert, fine-grained quartzarenite, and radiolarite, with limestone also present. Local sources primarily consisted of pebble deposits from the nearby riverbeds. The production strategy employed was the Levallois, predominantly using direct percussion. Cores were extensively utilized across several production series, with meticulous preparation of the striking platform by faceting and arranging the lateral and distal convexities using debordant flakes. Initially, production was unidirectional and convergent, later shifting towards centripetal and finally to preferential modalities. This strategy facilitated the production of standardized Levallois blanks, including both flakes and blades. Among the retouched tools, scrapers, long scrapers, and points were predominantly manufactured. These tools were often made from blanks that were already targeted in the debitage process ^[1]^ (Marciani study on going).

In the Uluzzian lithic assemblage the primary raw materials include chert, quartzarenite, and occasionally radiolarite sourced from nearby riverbeds, as well as limestone from the cave’s walls. The inherent characteristics (i.e., size and shape) of the original blocks, were crucial in determining the sizes and types of the final products ^[2]^. The bipolar technique is often utilized in conjunction with direct percussion. Anvils were made from compact, elastic rocks chosen for their ability to rebound force, and, sometimes, modified to fit into supports for added stability ^[3]^. The production strategy started from unmodified natural surfaces and used cores arranged as parallel planes, orthogonal planes, and semi-tournant, leading to less standardized artifacts than other methods like Levallois or volumetric blade reduction. This resulted in a diverse range of product shapes, sizes, and angles, including large, thick and thin flakes, flakelets, and bladelets^4^ (Marciani et al., submitted). Retouched tools, specifically designed for targeted functions such as lunates for projectiles and endscrapers for hide working, were crafted from meticulously selected blanks to meet specialized needs ^[5]^. In contrast, general-purpose tools like scrapers and points were produced from blanks with pre-existing functional shapes and underwent minimal retouching to effectively utilize their inherent features ^[4]^ (Marciani et al., submitted). Bone tools are also present ^[1]^. Interestingly the study of bird remains from the Uluzzian layers has revealed the occurrence of cutmarks due to the removal of feathers from the wing bones of a Eurasian hobby (*Falco subbuteo*), a Yellow-billed Chough (*Pyrrhocorax graculus)* and a large-sized Accipitridae (probably a Eurasian griffon), possibly to be connected with the use of fletching technology ^[6]^.

In both the Protoaurignacian (layer *rsa’*) and the Early Aurignacian (layers *gic* and *ars*), knappers primarily utilized fine-grained chert sourced near the site and imported as blocks and flakes. Minor frequencies of other raw materials, such as quartzite, radiolarite, and limestone, were also utilized, primarily in the production of flakes. Unlike the preceding Uluzzian layers, direct freehand percussion is the prevailing method for detaching blanks, although bipolar percussion on anvil continued to be employed throughout the *rsa’*–*ars* sequence, especially in the production of flakes. Bladelet production stands out as the most defining feature of Aurignacian assemblages at Castelcivita. The key distinction between the Protoaurignacian and the Early Aurignacian lies in their core types: prismatic cores were characteristic of the former, while carinated cores were prevalent in the latter ^[7]^. These differing reduction strategies resulted in the production of blanks of varying sizes and shapes. Notably, the Early Aurignacian is distinguished by the production of miniaturized bladelets with a stouter morphology compared to those of the Protoaurignacian. In both cultural variants, retouched bladelets dominate as the primary tool type, although there are noticeable differences between them. In the Protoaurignacian, long and straight bladelets (i.e., Dufour bladelets) were predominantly modified using inverse marginal retouching. Conversely, in the Early Aurignacian, the smallest bladelets were selected and modified with direct, often bilateral, retouching to achieve a pointed distal end. General-purpose tools are comparatively less common and are primarily produced from flakes and secondarily blades. Endscrapers are the most frequently encountered general-purpose tools in the Early Aurignacian, while retouched flakes and blades are more prevalent in the Protoaurignacian. Throughout the Aurignacian sequence, endscrapers consistently outnumber burins. Bone tools and several ornaments on marine shells were retrieved, especially in the Early Aurignacian layers.

References

^1^Gambassini, P. Le industrie paleolitiche di Castelcivita. In: Gambassini, P. (Ed.), *Il Paleolitico di Castelcivita, cultura e ambiente*. Napoli, Electa Napoli, 92–145. (1997).

^2^Marciani, G., Carmignani, L., Djakovic, I., Roussel, M., Arrighi, S., Rossini, M., Boschin, F., Ronchitelli, A., Benazzi, S., Moroni, A., & Soressi, M. (2025). The Uluzzian and Châtelperronian: No Technological Affinity in a Shared Chronological Framework. *Journal of Paleolithic Archaeology*, 8(1), 3. <https://doi.org/10.1007/s41982-024-00202-1>

^3^Arrighi, S. *et al.* Between the hammerstone and the anvil: bipolar knapping and other percussive activities in the late Mousterian and the Uluzzian of Grotta di Castelcivita (Italy). *Archaeol Anthropol Sci* **12**, 271. <https://doi.org/10.1007/s12520-020-01216-w> (2020).

^4^Rossini, M. et al. Less is more! Uluzzian technical behaviour at the cave site of Castelcivita (southern Italy). *Journal of Archaeological Science: Reports* **44**, 103494. <https://doi.org/10.1016/j.jasrep.2022.103494> (2022).

^5^Sano, K. et al. The earliest evidence for mechanically delivered projectile weapons in Europe. Nat Ecol Evol 3, 1409–1414 <https://doi.org/10.1038/s41559-019-0990-3> (2019).

^6^Fiore, I. *et al.* Archeozoology and taphonomy of bird remains from Grotta di Castelcivita (Salerno, Italy) and clues for human-bird interactions. *Quaternary International* **551**, 224–242. <https://doi.org/10.1016/j.quaint.2019.09.004> (2020).

^7^Falcucci, A. *et al.* A pre-Campanian Ignimbrite techno-cultural shift in the Aurignacian sequence of Grotta di Castelcivita, southern Italy. *Sci Rep* **14**, 12783. <https://doi.org/10.1038/s41598-024-59896-6> (2024).

Table S1: Morphologically identified macrofauna grouped into categories comparable to the ZooMS identified material (data from Masini and Abbazzi 1997 and Fiore et al. 2020).

| **Faunal Categories** | **ZooMS**  **Identification** | **Taxa** | **Mousterian**  **NISP** | **Uluzzian**  **NISP** | **Protoaurignacian**  **NISP [RSA’ only]** | | **Total** |
| --- | --- | --- | --- | --- | --- | --- | --- |
| **Equid** | *Equus* | *Equus ferus* | 3 | 73 | 11 | [10] | 87 |
| **Cervidae** | Cervid | *Cervus elaphus* | 107 | 49 | 32 | [6] | 188 |
|  |  | *Dama dama* | 137 | 20 | 2 | [1] | 159 |
| **Cervidae** | Capreolus capreolus | *Capreolus capreolus* | 29 | 34 | 10 | [2] | 73 |
| **Cervidae** | Cervid | *Megaloceros giganteus* | 0 | 1 | 0 | [0] | 1 |
| **Bos/Bison** | *Bos*/*Bison* | *Bison priscus* | 10 | 9 | 7 | [6] | 26 |
|  |  | *Bos*/*Bison* | 4 | 8 | 0 | [0] | 12 |
| **Caprine** | *Capra* sp. | *Capra ibex* | 53 | 12 | 4 | [1] | 69 |
| **Caprine** | *Rupicapra* sp. | *Rupicapra* sp. | 85 | 25 | 13 | [0] | 123 |
| **Rhinoceros** | Rhinoceros | *Stephanorhinus* sp. | 1 | 0 | 0 | [0] | 1 |
| **Cervidae** | Unidentified Cervid | Cervidae indet. | 17 | 5 | 3 | [0] | 25 |
| **-** | Unidentified Caprines | **-** | - | - | - | - | - |
| **-** | Artiodactyla | **-** | - | - | - | - | - |
| ***Sus* sp.** | *Sus* sp. | *Sus scrofa* | 7 | 8 | 11 | [7] | 26 |
| **Carnivore** | *Ursus* sp. | *Ursus spelaeus* | 1 | 1 | 0 | [0] | 2 |
|  |  | *Ursus arctos* | 0 | 4 | 0 | [0] | 4 |
| **Carnivore** | Canid | *Canis lupus* | 0 | 1 | 2 | [0] | 3 |
|  |  | *Vulpes vulpes* | 0 | 4 | 0 | [0] | 4 |
| **Carnivore** | *Felis*/*Lynx*/*Ursus* | *Felis silvestris* | 0 | 5 | 0 | [0] | 5 |
| **Carnivore** | *Panthera*/*Crocuta* | *Panthera pardus* | 4 | 5 | 0 | [0] | 9 |
|  |  | *Panthera leo spel.* | 0 | 0 | 0 | [0] | 0 |
|  |  | *Crocuta crocuta spelaea* | 11 | 7 | 1 | [0] | 19 |
| **Carnivore** | Unidentified Carnivore | Carnivora indet*.* | 11 | 9 | 2 | [0] | 22 |
|  |  | *Mustela nivalis* | 1 | 3 | 0 | [0] | 4 |
|  |  | *Martes* sp. | 0 | 0 | 0 | [0] | 0 |
|  |  | *Meles meles* | 0 | 2 | 0 | [0] | 2 |
|  |  | Mustelidae | 0 | 2 | 0 | [0] | 2 |
|  | Total |  | 481 | 287 | 98 | [33] | 866 [801] |

Table S2: ZNISP values and Morphological NISP values combined into broad faunal categories to compare between the 3 cultural complexes.

|  | **Mousterian** | | **Uluzzian** | | **Protoaurignacian** | |
| --- | --- | --- | --- | --- | --- | --- |
| **Faunal Category** | ZNISP | NISP | ZNISP | NISP | ZNISP | NISP |
| Equid | 3 | 3 | 338 | 73 | 107 | 10 |
| *Bos*/*Bison* | 13 | 14 | 96 | 17 | 47 | 6 |
| Caprine | 30 | 138 | 69 | 37 | 11 | 1 |
| Cervidae | 134 | 290 | 209 | 109 | 24 | 9 |
| Carnivore | 6 | 28 | 6 | 43 | 1 | 0 |
| Rhinoceros | 2 | 1 | 0 | 0 | 0 | 0 |
| *Sus* sp. | 2 | 7 | 13 | 8 | 0 | 7 |
| Artiodactyla | 2 | - | 1 | - | 0 | - |
| **Total** | 192 | 481 | 732 | 287 | 190 | 33 |

Table S3: Morphological macrofaunal identifications, originally published in Masini and Abbazzi 1997.

| **Cultural Layer** | **SPIT** | **LAYER** | | ***Equus*** | ***Stephanorhinus* sp.** | ***Sus scrofa*** | ***Bison priscus*** | ***Bos/Bison*** | ***Capra ibex*** | ***Rupicapra* sp.** | ***Megaloceros giganteus*** | ***Cervus elaphus*** | ***Dama dama*** | ***Capreolus capreolus*** | **Cervidae indet.** | ***Ursus arctos*** | ***Ursus spelaeus*** | ***Canis lupus*** | ***Vulpes vulpes*** | ***Panthera pardus*** | ***Lynx lynx*** | ***Felis silvestris*** | ***Crocuta crocuta spelaea*** | ***Meles meles*** | ***Mustela nivalis*** | **Mustelidae** | **Carnivora indet.** |
| --- | --- | --- | --- | --- | --- | --- | --- | --- | --- | --- | --- | --- | --- | --- | --- | --- | --- | --- | --- | --- | --- | --- | --- | --- | --- | --- | --- |
| Proto Aurig. |  | ars |  |  |  |  |  |  |  | 2 |  | 3 |  | 1 |  |  |  |  |  |  |  |  |  |  |  |  |  |
|  |  | gic |  | 1 |  | 4 | 1 |  | 3 | 11 |  | 23 | 1 | 7 | 3 |  |  | 2 |  |  |  |  | 1 |  |  |  | 2 |
|  |  | RSA' | DA | 1 |  | 3 | 1 |  |  |  |  | 4 | 1 | 2 |  |  | 1 |  |  |  |  |  | 2 |  |  |  |  |
|  |  |  | DB | 9 |  | 4 | 5 |  | 1 |  |  | 2 |  |  |  |  |  | 1 | 1 |  |  | 1 | 3 |  | 1 |  |  |
| Uluzzian | 10 | RSA'' | UA | 16 |  |  | 1 |  |  |  | 1 | 2 |  | 1 |  |  |  |  | 1 |  |  |  | 1 |  |  |  |  |
|  | 11 |  | UB | 17 |  | 1 | 2 | 1 | 1 |  |  | 6 | 3 | 2 | 1 |  |  |  |  | 2 |  | 2 |  |  |  |  |  |
|  | 12 |  | UC | 20 |  | 1 | 2 | 2 | 2 |  |  | 2 |  | 1 |  |  |  |  |  |  |  |  |  |  | 1 |  | 1 |
|  | 12-13 | RPI | UD | 12 |  | 2 |  | 1 | 3 | 1 |  | 5 |  | 1 |  | 2 |  |  |  |  | 1 |  |  |  | 1 | 1 |  |
|  | 13 |  | UE |  |  | 2 |  |  | 1 | 4 |  | 12 |  | 1 |  | 1 |  |  |  |  |  |  | 1 |  |  |  |  |
|  | 14 | PIE | UF | 2 |  |  |  |  |  | 2 |  | 5 | 5 | 2 |  |  |  |  |  | 1 |  |  |  |  |  |  |  |
|  | 15 |  | UG | 3 |  | 2 | 1 | 2 | 2 | 5 |  | 11 | 4 | 8 | 2 |  |  |  | 1 | 1 |  |  |  | 1 |  |  | 4 |
|  |  |  | UH | 1 |  |  | 1 | 1 | 2 | 6 |  | 4 | 1 | 11 | 1 |  |  |  | 1 | 1 |  | 1 |  | 1 |  | 1 | 2 |
|  | 16 | RSI | UI | 1 |  |  | 1 |  |  | 2 |  | 1 | 6 | 2 |  |  |  |  |  |  |  | 1 |  |  |  |  | 1 |
|  | 17 |  | UL | 1 |  |  | 1 | 1 | 1 | 5 |  | 1 | 1 | 5 | 1 |  |  |  |  |  |  |  |  |  |  |  | 1 |
|  | 18 |  | MA18 |  |  |  |  |  |  | 2 |  | 1 | 2 |  |  |  |  |  |  | 1 |  |  |  |  |  |  |  |
| Mous. | 19 |  | MA |  |  |  |  | 2 | 2 | 20 |  | 5 | 5 | 5 |  | 1 | 1 |  |  |  |  |  |  |  |  |  | 2 |
|  | 20 |  | MB | 1 |  |  | 1 |  |  | 12 |  | 7 | 2 | 7 | 1 |  |  |  |  |  |  |  |  |  |  |  | 2 |
| Mousterian | 21 |  | MC |  |  |  |  | 2 | 2 | 3 |  | 1 | 3 | 1 | 7 |  |  |  |  |  |  |  |  |  |  |  | 1 |
|  | 21-22 | GAR | gar1 |  | 1 |  |  |  | 1 | 3 |  | 11 | 2 | 2 |  |  |  |  |  |  |  |  | 3 |  |  |  | 1 |
|  | 23-24 |  | gar2 | 1 |  | 1 |  |  | 1 | 2 |  | 5 | 9 | 2 |  |  |  |  |  |  |  |  | 5 |  |  |  | 1 |
|  | 25 |  | gar3 |  |  |  |  |  | 2 | 1 |  | 6 | 4 | 1 |  |  |  |  |  |  |  |  |  |  |  |  |  |
|  | 26 |  | gar4 | 1 |  |  |  |  | 1 | 1 |  | 1 | 4 | 1 | 1 |  |  |  |  |  |  |  |  |  |  |  |  |
|  | 27 | CGR | gar5 |  |  |  | 3 |  | 8 | 5 |  | 17 | 5 | 3 | 1 |  |  |  |  | 1 |  |  |  |  |  |  | 1 |
|  | 28 |  | gar6 |  |  | 4 | 2 |  | 13 | 9 |  | 16 | 24 | 3 |  |  |  |  |  |  |  |  | 1 |  |  |  | 1 |
|  | 29 |  | gar7 |  |  |  | 2 |  | 10 | 2 |  | 9 | 26 |  | 1 |  |  |  |  | 1 |  |  | 1 |  |  |  |  |
|  | 30 |  | gar8 |  |  |  | 1 |  | 6 | 15 |  | 17 | 23 | 1 | 3 |  |  |  |  | 1 |  |  |  |  |  |  | 1 |
|  | 31 |  | gar9 |  |  | 1 |  |  | 6 | 8 |  | 9 | 24 | 1 |  |  |  |  |  |  |  |  | 1 |  |  |  | 1 |
|  | 32-33 |  | gar10 |  |  | 1 | 1 |  | 1 | 2 |  | 2 | 4 | 2 | 3 |  |  |  |  |  |  |  |  |  | 1 |  |  |
|  | | | Total NISP | 87 | 1 | 26 | 26 | 12 | 69 | 123 | 1 | 188 | 159 | 73 | 25 | 4 | 2 | 3 | 4 | 9 | 1 | 5 | 19 | 2 | 4 | 2 | 22 |
|  |  |  | NMI | 39 | 1 | 16 | 18 | 12 | 46 | 73 | 1 | 98 | 81 | 44 | 16 | 3 | 2 | 3 | 4 | 8 | 1 | 5 | 15 | 2 | 4 | 2 | 20 |

Table S4: ZooMS Ids separated by square and layer (Oertle samples only n=968).

| **Square** | | **Layer** | **Fail** | ***C. capreolus*** | ***Equus*** | **Cervid/*Equus*** | **Cervid** | ***Capra* sp.** | **Cervid/Chamois** | **Uniden. Cervid** | **Uniden. Caprines** | **Chamois** | ***Bos*/*Bison*** | **Artiodactyla** | **Rhinoceros** | ***Sus* sp.** | ***Panthera*/*Crocuta*** | ***Ursus* sp.** | **Canina** | ***Felis*/*Lynx*/*Ursus*** | **Carnivore** | **Success Total** | **Square Total** |
| --- | --- | --- | --- | --- | --- | --- | --- | --- | --- | --- | --- | --- | --- | --- | --- | --- | --- | --- | --- | --- | --- | --- | --- |
| **E12** | E12III | PIE |  | 1 | 4 |  | 3 |  |  |  |  | 4 | 4 |  |  |  |  |  | 1 |  |  | 17 | 17 |
|  | E12II | RPI | 1 |  |  |  | 2 |  |  |  |  |  |  |  |  |  |  |  |  |  |  | 2 | 3 |
| **E13** | E13 I-II | GAR |  |  |  |  | 7 |  |  |  |  | 1 |  |  |  |  |  |  |  |  |  | 8 | 8 |
|  | E13IV | GAR |  |  |  |  |  | 1 |  | 2 |  | 1 |  |  |  |  |  | 1 |  |  |  | 5 | 5 |
| **E14** | E14 I-II | GAR |  |  |  |  | 6 |  |  | 4 |  | 1 | 2 |  |  |  |  |  |  |  |  | 13 | 13 |
|  | E14 I/IV | RSA' | 3 |  | 7 |  | 2 |  |  |  |  | 1 | 4 |  |  |  |  |  |  |  |  | 14 | 17 |
|  | E14 I/IV | RSA'' |  |  | 3 |  | 1 | 1 |  |  |  |  |  |  |  |  |  |  |  |  |  | 5 | 5 |
| **F12** | F12 | PIE |  | 6 | 1 |  | 16 | 1 |  |  |  | 3 | 1 |  |  |  |  |  |  |  | 2 | 30 | 30 |
| **F13** | F13 III-IV | GAR |  |  |  |  | 11 |  |  | 2 |  | 1 |  |  |  |  |  | 1 |  | 1 |  | 16 | 16 |
|  | F13 I-II | GAR | 2 |  |  |  | 8 |  |  |  |  |  | 1 |  |  |  |  |  |  |  |  | 9 | 11 |
|  | F13 I-II | CGR | 4 |  |  |  | 5 |  |  | 4 |  |  | 1 |  |  |  |  |  |  |  |  | 10 | 14 |
| **F14** | F14 II/F14 III-IV | GAR | 2 |  |  |  | 23 |  |  |  |  |  | 2 |  |  |  |  |  |  |  |  | 25 | 27 |
|  | FI4II | PIE | 1 |  |  |  | 5 |  |  |  |  |  | 4 |  |  |  |  |  |  |  |  | 9 | 10 |
|  | F14IV | RPI |  |  | 4 |  |  |  |  |  |  |  | 2 |  |  | 1 |  | 1 |  |  |  | 8 | 8 |
|  | F14 | RSA' | 3 |  | 19 |  | 4 |  |  |  |  | 1 | 2 |  |  |  |  |  |  |  |  | 26 | 29 |
|  | F14 I | RSA'' | 1 |  |  |  |  |  |  |  |  |  |  |  |  |  |  |  |  |  |  | 0 | 1 |
| **G12** | G12II | PIE |  | 2 |  |  | 8 |  |  |  |  | 1 | 2 |  |  | 1 |  | 1 |  |  | 1 | 16 | 16 |
|  | G12 I | GAR | 1 |  | 1 |  | 14 | 2 |  |  |  | 1 |  |  |  | 2 |  |  |  | 1 |  | 21 | 22 |
|  | G12I | RPI |  |  | 10 |  | 4 |  |  |  | 1 |  | 2 |  |  | 1 |  |  |  |  |  | 18 | 18 |
| **G13** | G13 | CGR | 1 | 1 |  |  | 7 |  | 2 | 1 |  | 1 |  |  |  |  |  |  |  |  |  | 12 | 13 |
|  | G13 I-II | GAR | 7 |  | 1 |  | 34 | 11 |  | 2 | 1 |  | 5 |  | 1 |  |  | 1 | 1 |  |  | 57 | 64 |
|  | G13 | PIE |  |  |  |  | 5 | 1 |  |  |  |  | 2 |  |  |  |  |  |  |  |  | 8 | 8 |
|  | G13II | RPI | 5 |  | 15 |  | 13 | 2 |  |  |  |  | 3 |  |  |  |  |  |  |  |  | 33 | 38 |
|  | G13 | RSA' | 1 |  | 10 |  | 3 |  |  |  |  | 1 | 6 |  |  |  |  |  |  |  |  | 20 | 21 |
| **G14** | GI4II | GAR | 1 |  | 1 |  | 3 | 7 |  |  |  | 2 | 2 |  | 1 |  |  |  |  |  |  | 16 | 17 |
|  | G14II | PIE |  |  |  |  | 4 |  |  |  |  |  |  |  |  |  |  |  |  |  |  | 4 | 4 |
|  | G14II - III | RPI | 3 | 1 | 21 |  | 3 |  |  | 3 | 1 |  | 3 |  |  | 3 |  |  |  |  |  | 35 | 38 |
|  | G14 I , II, IV | RSA'' | 1 |  | 19 | 1 | 1 |  |  |  |  |  | 6 |  |  |  |  |  |  |  |  | 27 | 28 |
|  | G14 , III | RSA' | 7 |  | 8 |  | 2 | 1 |  |  | 2 | 2 | 9 |  |  |  | 1 |  |  |  |  | 25 | 32 |
| **H12** | H12I, 11+III | RPI |  |  | 6 |  | 1 |  |  |  |  |  | 1 |  |  |  |  |  |  |  |  | 8 | 8 |
|  | H12I, IV | RSA' | 3 |  | 15 |  | 2 |  |  |  |  |  | 6 |  |  |  |  |  |  |  |  | 23 | 26 |
|  | H12 I | RSA'' | 1 |  | 16 |  | 2 |  |  | 1 |  |  | 5 |  |  |  |  |  |  |  |  | 24 | 25 |
| **H13** | H13IV | PIE | 7 |  |  |  | 2 |  |  |  |  | 1 |  | 1 |  |  |  |  |  |  |  | 4 | 11 |
|  | H13II | RPI | 20 | 1 | 11 |  | 1 | 2 |  |  | 1 | 1 |  |  |  |  |  |  |  |  |  | 17 | 37 |
|  | H13II | RSA' | 2 |  | 20 |  | 6 | 3 |  |  |  |  | 10 |  |  |  |  |  |  |  |  | 39 | 41 |
|  | H13 II | RSA'' | 23 |  | 36 |  | 8 | 2 |  | 1 |  |  | 12 |  |  |  |  |  |  |  |  | 59 | 82 |
| **H14** | H14II | PIE | 7 | 1 | 1 |  | 6 | 1 |  | 1 |  | 16 | 1 |  |  |  |  |  |  |  |  | 27 | 34 |
|  | H14III | RPI | 15 |  | 20 |  | 7 | 1 |  |  |  |  |  |  |  | 2 |  |  |  |  |  | 30 | 45 |
|  | H14IV | RSA' | 3 |  | 28 |  | 4 |  |  | 1 |  |  | 10 |  |  |  |  |  |  |  |  | 43 | 46 |
|  | H14 II | RSA'' | 18 |  | 26 |  | 15 |  |  | 1 |  | 2 | 18 |  |  |  |  |  |  |  |  | 62 | 80 |
| **Total** | | | 143 | 13 | 303 | 1 | 248 | 36 | 2 | 23 | 6 | 41 | 126 | 1 | 2 | 10 | 1 | 5 | 2 | 2 | 3 | 825 | 968 |

Table S5: Radiocarbon dates for the site of Castelcivita from Higham et al. 2024.

** denotes duplicate results from the same bone, but with different methods.

In the pretreatment method column, AF = samples treated using ultrafiltration of collagen. HYP = a sample dated using compound-specific Hydroxyproline dating. YR = a sample dated using the AOx-SC method. XR = a sample dated using the ABOx-SC method. Error terms are reported at ± 1 sigma. OxA-22622 was previously published by Douka et al. (2014). Taglio is the Italian term for spit, a pre-determined unit of stratigraphic depth used in excavation. These results were originally published in Higham et al. (2024).

| OxA | Material | Layer/taglio/square | Industry | Species/ZooMS id | Pretreatment Method | ^14^C age BP | ± error |
| --- | --- | --- | --- | --- | --- | --- | --- |
| X-2698-45 | charcoal | Sample 1 Focolare L11/12 | Aurignacian | Not identified | YR | 34,380 | 310 |
| 22622 | charcoal | RSA”, spit 11 | Uluzzian | c.f. *Ilex aquifolium* | XR | 36,120 | 360 |
| X-2770-41 | bone | RSA" lower 11 F14II | Uluzzian | Cervid | AF | 38,300 | 1,000 |
| X-2770-40 | bone | RSA" lower 11 F14II | Uluzzian | Equid | AF | 38,300 | 1,000 |
| X-2770-39 | bone | RSA" lower 11 F14IV | Uluzzian | Equid | AF | 38,000 | 900 |
| 37247 | bone | RSA" Upper 12 F14I | Uluzzian | Cervid | AF | 37,500 | 900 |
| 37246 | bone | RSA" Upper 12 F14I | Uluzzian | Cervid | AF | 38,500 | 1,000 |
| 39604 | bone | RSA" Taglio 12 upper G14IV | Uluzzian | Equid | AF | 36220 | 750 |
| 37245 | bone | RSA" Upper 12 G14IV | Uluzzian | Rupicapra | AF | 37,800 | 900 |
| X-2733-13** | bone | RPI Taglio 12 low G13 II | Uluzzian | Bovidae/Cervidae/ Giraffidae | AF | 36,000 | 700 |
| X-2772-7** | bone | RPI Taglio 12 low G13 II | Uluzzian | Bovidae/Cervidae/ Giraffidae | HYP | 35,100 | 1,100 |
| X-2698-46 | charcoal | Base RSA" | Uluzzian | Not identified | YR | 36,480 | 390 |
| 37251 | bone | RPI Taglio 13 G13 | Uluzzian | Bovidae | AF | 37,400 | 900 |
| 37250 | bone | RPI Taglio 13 G13 | Uluzzian | Bovidae | AF | 38,700 | 1,000 |
| 39605 | bone | RPI Upper 14 G13II | Uluzzian | Cervid | AF | 37,730 | 910 |
| 37244 | bone | Upper 14 RPI G14II | Uluzzian | Cervid | AF | 39,300 | 1,100 |
| 37243 | bone | Upper 14 RPI G14II | Uluzzian | Cervid | AF | 38,600 | 1,000 |
| 37252 | bone | RPI Upper 14 G13II | Uluzzian | Cervid | AF | 39,100 | 1,100 |
| 37254 | bone | PIE Lower 15 - Upper 16 H14I | Uluzzian | Equid | AF | 38,600 | 1,000 |
| 37253 | bone | PIE Lower 15 - Upper 16 H14I | Uluzzian | Cervid | AF | 38,600 | 1,100 |
| 37249 | bone | RSI' 16 lower H13I | Uluzzian | Rupicapra | AF | 38,500 | 1,000 |
| 37248 | bone | RIS' 16 lower H13I | Uluzzian | Capra | AF | 38,500 | 1,000 |
| X-2733-15 | bone | RSI Taglio 19 low H14 III | Mousterian | Deer/Gazelle/Saiga | AF | 40,500 | 1,200 |
| X-2733-14 | bone | RSI Taglio 20 low F14 II | Mousterian | Ovidae/Cervidae/  Giraffidae | AF | 38,800 | 1,000 |
| X-2733-12 | bone | RSI Taglio 20 low F12 I | Mousterian | Ovis/Capra | AF | 39,600 | 1,100 |

Table S6. OSL doses and ages of the sediment samples from Castelcivita.

^a^ ‘noxford’ is the number of aliquots measured at the Oxford laboratory, ‘nstonybrook’ is the number of aliquots measured at the Stony Brook laboratory.

b Equivalent dose (De) and overdispersion (OD) were calculated using the central age model (Galbraith et al., 1999).

c Ages are reported in 10^3^ years (ka) and the uncertainty shown after the ± symbol is the quadratic sum of the random and systematic uncertainties at 1σ; values shown in brackets are the random-only errors (used for Bayesian modelling purposes).

P = Protoaurignacian U = Uluzzian M = Mousterian

Originally published in Higham et al., (2024).

| **Sample** **ID** | **Layer** | **nOxford** **+** **nStonyBrook**  **a** | **De** **(Gy)^b^** | **OD** **(%)^b^** | **OSL** **age** **(ka)^c^** |
| --- | --- | --- | --- | --- | --- |
| CTC_X7021_SB4 | ARS(P) | 6+7 | 131.88 ± 9.18 | 19 ± 4 | 36.7 ± 3.3 (2.8) |
| CTC_X7022_SB5 | ARS(P) | 3+12 | 112.89 ± 5.88 | 16 ± 3 | 38.9 ± 2.9 (2.2) |
| CTC_X7023_SB6 | RSA’(P) | 3+5 | 123.81 ± 7.70 | 16 ± 4 | 42.4 ± 3.7 (3.0) |
| CTC_X7024_SB7 | RSA’(P)/RSA’’( | 3+9 | 121.98 ± 4.22 | 10 ± 2 | 42.4 ± 2.8 (2.0) |
| CTC_X7025_SB8 | U) | 10+5 | 104.84 ± 5.83 | 16 ± 3 | 37.9 ± 2.9 (2.2) |
| CTC_X7026_SB9 | RPI(U) | 5+6 | 110.89 ± 8.07 | 21 ± 5 | 39.5 ± 3.5 (3.0) |
| CTC_X7027_SB10 | RPI/PIE(U) | 0+12 | 114.21 ± 3.01 | 8 ± 2 | 41.8 ± 2.4 (1.3) |
| CTC_X7028_SB11 | PIE/RSI(U) | 3+9 | 105.34 ± 6.07 | 19 ± 5 | 39.2 ± 3.1 (2.4) |
| CTC_X7029_SB12 | RSI(U)/RSI(M) | 4+27 | 97.03 ± 5.70 | 31 ± 4 | 47.0 ± 3.7 (3.0) |
| CTC_X7030_SB13 | RSI(M) | 4+19 | 127.48 ± 4.13 | 14 ± 2 | 48.6 ± 3.3 (2.1) |
|  | GAR(M) |  |  |  |  |

**Statistical tests on ZooMS and Morph values for each period. Tests undertaken in Past 4.03 (Hammer et al. 2001).**

H0: ZooMS NISP values are independent of Morphological NISP values.

Mousterian – Chi^2^ = 22.178 p=0.001 DF=6

Uluzzian - Chi^2^ = 126.8 p=1.1359E-25 DF=5

Protoaurignacian - Chi^2^ = 49.082 p=2.1353E-09 DF=5

All p values are <0.05 therefore the null hypothesis is rejected and conclude that there is an association between ZooMS and Morph NISP values.


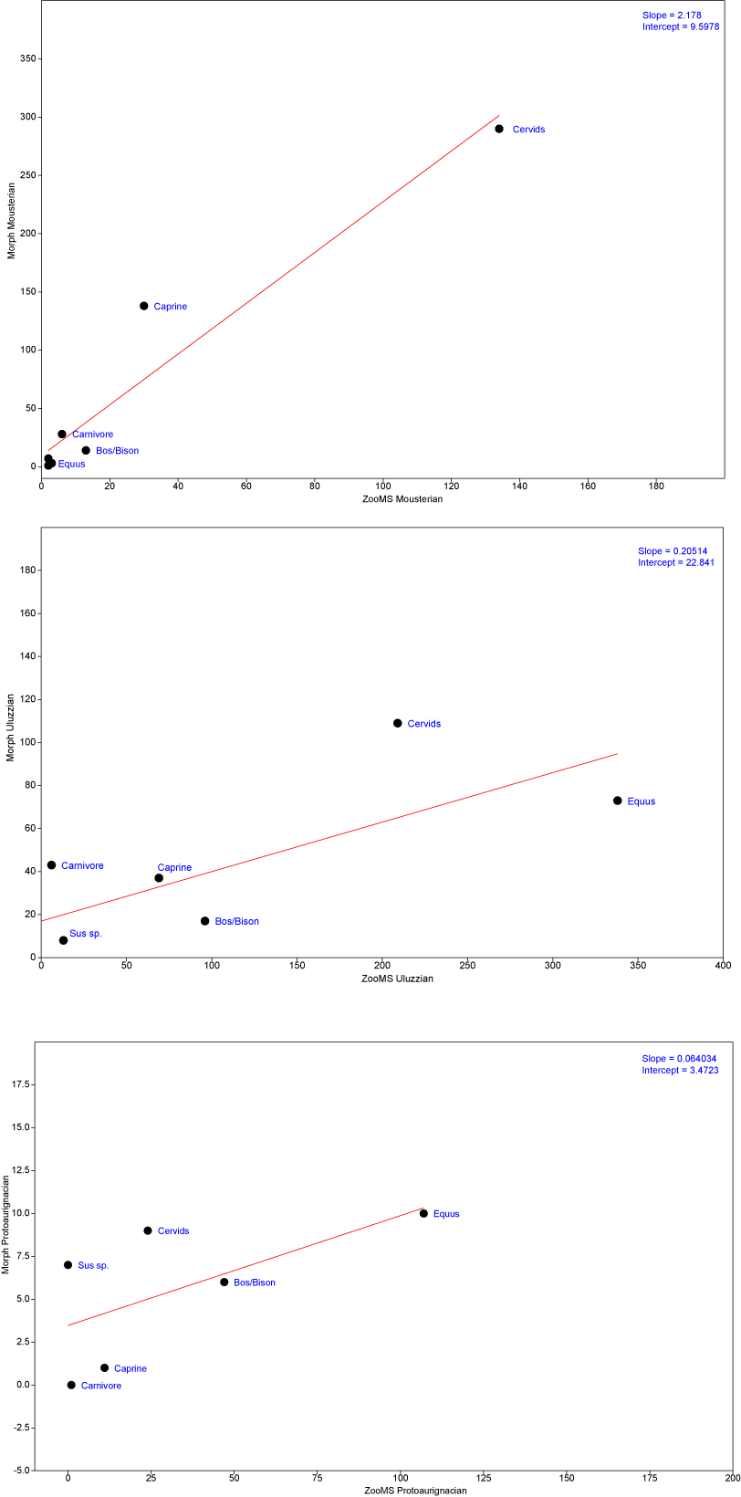


Figure S1: Bivariate linear regression plots for each period. All showing positive correlation between Morph and ZooMS values. The Mousterian and Protoaurignacian plots show the 1^st^ ranked taxon remain so for both ZooMS and Morph, whilst the Uluzzian shows a shift from Cervids in the Morph to Equus in the ZooMS.

| **# of markers** | **# of ID samples** | **% of successful IDs** | **CGR** | **GAR** | **PIE** | **RPI** | **RSA"** | **RSA'** |
| --- | --- | --- | --- | --- | --- | --- | --- | --- |
| **4** | 5 | 1 |  |  |  | 1 | 2 | 2 |
| **5** | 17 | 2 | 2 | 8 | 2 | 3 | 2 |  |
| **6** | 24 | 2 | 1 | 3 | 4 | 3 | 8 | 5 |
| **7** | 48 | 5 | 6 | 7 | 3 | 6 | 13 | 13 |
| **8** | 131 | 14 | 6 | 7 | 16 | 39 | 43 | 20 |
| **9** | 600 | 62 | 7 | 145 | 90 | 99 | 109 | 150 |
| **Total** | **825** | **85** | **22** | **170** | **115** | **151** | **177** | **190** |

Table S7: The total number of peptide markers that were successfully taxonomically identified over the different stratigraphic layers, with the total number of ZooMS samples = 968. Only samples with 4 or more markers were deemed successful with 825/968 samples and an 85% success rate. No samples from *rsi* are recorded for the Oertle processed samples.

Table S8: Percentage of samples with the number of markers present in each stratigraphic layer. Colour scale to represent the variation of preservation between different layers. The maximum number of markers that could be present is 9.

| **# markers** | **CGR** | **GAR** | **PIE** | **RPI** | **RSA"** | **RSA'** |
| --- | --- | --- | --- | --- | --- | --- |
| **4** | 0% | 0% | 0% | 1% | 1% | 1% |
| **5** | 9% | 5% | 2% | 2% | 1% | 0% |
| **6** | 5% | 2% | 3% | 2% | 5% | 3% |
| **7** | 27% | 4% | 3% | 4% | 7% | 7% |
| **8** | 27% | 4% | 14% | 26% | 24% | 11% |
| **9** | 32% | 85% | 78% | 66% | 62% | 79% |
